# TIM-4^+^ skeletal muscle Resident Tissue Macrophages Ferroptosis mediated Rhabdomyolysis in Exertional Heatstroke

**DOI:** 10.7150/ijbs.114815

**Published:** 2026-03-17

**Authors:** Youyong Tang, Qiyuan An, Keying Zhang, Chenxin Liu, Riqing Wei, Ru Li, Zhiqing Wang, Sixiao He, Fudi Wang, Li Ma, Qiang Ma

**Affiliations:** 1 Department of Clinical Laboratory, Shandong Provincial Hospital Affiliated to Shandong First Medical University, Jinan 250021, Shandong, China.; 2 Department of Biopharmaceutics, School of Laboratory Medicine and Biotechnology, Southern Medical University, Guangzhou, 510515, China.; 3 Henan Red Cross Blood Center, Zhengzhou, 450004, China.; 4 School of Medicine, The Chinese University of Hong Kong, Shenzhen, 518172, China.; 5 The Second Affiliated Hospital, School of Public Health, State Key Laboratory of Experimental Hematology, Zhejiang University School of Medicine, Hangzhou 310058, China.; 6 Global Innovation Institute of Element Science (GIIES-JLU), The First Hospital of Jilin University, Changchun 130021, China.; 7 Institute of Molecular Immunology, School of Laboratory Medicine and Biotechnology, Southern Medical University, Guangzhou, China.; 8 State Key Laboratory of Multi-organ Injury Prevention and Treatment, Southern Medical University, Guangzhou, China.; 9 Key Laboratory of Infectious Diseases Research in South China, Ministry of Education, Southern Medical University, Guangzhou, China.; 10 Key Laboratory of Extreme Environmental Medicine, Ministry of Education of China, Chongqing 400038, China.

**Keywords:** Ferroptosis, T cell membrane protein 4-positive skeletal muscle resident tissue macrophages, olfactory receptor 2, NOD-like receptor family pyrin domain containing 3, Rhabdomyolysis.

## Abstract

Skeletal muscle resident tissue macrophages (smRTMs) are strategically positioned to sense myofiber injury and coordinate inflammatory responses, but their mechanistic contribution to exertional heatstroke (EHS)-associated rhabdomyolysis (RM) remains poorly defined. Here, we delineate a ferroptosis-dependent pathway in smRTMs that drives RM during EHS. Using mouse model of EHS, in combination with single-cell RNA sequencing, smRTM-targeted *Hmox1* deletion and pharmacological modulation of ferroptosis and inflammasome activity, we identify a T cell membrane protein 4-positive (TIM-4⁺) smRTM subset as selectively vulnerable to ferroptosis. EHS robustly induces heme oxygenase-1 (HMOX1), iron-dependent lipid peroxidation and ferroptotic death in TIM-4⁺ smRTMs, accompanied by accumulation of the lipid peroxidation-derived aldehyde octanal. Octanal engages olfactory receptor 2 (Olfr2) and provides a proximal signal for activation of the NOD-like receptor family pyrin domain containing 3 (NLRP3) inflammasome, caspase-1 cleavage and interleukin-1β (IL-1β) release. Chromatin and functional assays further establish JunD as a transcription factor that binds the *Olfr2* promoter and is required for *Olfr2* upregulation downstream of HMOX1-driven ferroptosis. Genetic or pharmacological inhibition of HMOX1, or blockade of ferroptosis, reduces TIM-4⁺ smRTM ferroptosis, dampens the JunD-Olfr2-NLRP3-IL-1β axis and significantly attenuates RM in both species. These data identify HMOX1-dependent ferroptosis in TIM-4⁺ smRTMs as a central immunometabolic mechanism of EHS-associated RM and nominate smRTM ferroptosis and the Olfr2-NLRP3 pathway as rational therapeutic targets.

## 1. Introduction

Rhabdomyolysis (RM) is a prevalent complication of exertional heatstroke (EHS) 1. It represents a clinical emergency caused by the rapid skeletal muscle breakdown, leading to the release of intracellular muscle components into the systemic circulation. This event has the potential to cause multiple organ injury and failure 1. Without prompt treatment, RM can result in significant morbidity and mortality induced by EHS 2. Nonetheless, the mechanism underlying RM following EHS remains unclear, and effective prevention and treatment methods are currently lacking.

The skeletal muscle tissue demonstrates some resistance to heat stress injury 3. In the majority of EHS patients, RM occurs after their body temperature has normalized, and even with aggressive cooling, it may not entirely prevent the progression of RM in patients with persistent hyperthermia 4, indicating that heat stress is not the exclusive factor leading to RM in EHS patients. Further research suggests that inflammation plays a crucial role in the occurrence and progression of RM 5. Skeletal muscle resident tissue macrophages (smRTM) are the initial responders in the inflammatory cascade when skeletal muscle tissue is injured 6, but their role in the progression of RM remains unclear.

Tissue-resident macrophages are renowned for their role as immune sentinels at the forefront of tissue defense. These cells express various receptors to recognize damage associated molecular patterns and quickly detect individual cell death 7. Following the initial recognition of death signals, tissue-resident macrophages release inflammatory cytokines and chemokines 8, driving inflammatory leukocytes (mainly neutrophils and monocytes) from the bloodstream into. Previous research indicates that Macrophage PM2.5 uptake triggers intracellular oxidant production, promoting the transmission of inflammatory signals and ultimately leading to the release of cytokines and chemokines 9. In chronic obstructive pulmonary disease, ferroptosis M2 alveolar macrophages (AMs) lose their anti-inflammatory and repair functions, instead provoking inflammatory responses, leading to sustained inflammation and tissue damage in conjunction with M1 AMs 10. This indicates that ferroptosis in tissue-resident macrophages can act as an amplifier, playing a crucial role in inflammatory responses.

Ferroptosis, a form of iron-dependent cell death, was first identified in cancer cells in 2012 by Dixon et al 11. Previous studies indicate that M1 microglia exhibit ferroptosis resistance compared with M2 microglia, attributable to NOS2-mediated nitric oxide (NO) production leading to reduced arachidonate 15-lipoxygenase (ALOX15) activity 12. Similarly, resident microenvironment of RTM undergoes continuous evolution influenced by the skeletal muscle tissue microenvironment, leading to a highly heterogeneous RTM phenotype with diverse functions 13. Importantly, recent studies have highlighted TIM-4⁺ tissue-resident macrophages as key regulators of tissue homeostasis and injury-associated remodeling, and have further demonstrated that ferroptosis can occur in resident macrophage populations under conditions of oxidative stress and iron dysregulation, thereby modulating their inflammatory potential 10, 14-19. However, to our knowledge, this is the first study demonstrating that ferroptosis of TIM-4⁺ RTMs actively promotes inflammatory responses, thereby linking RTM ferroptosis to muscle inflammation.

Several reports suggest that ferroptosis is accompanied by excessive generation of lipid peroxidation products, including 4-Hydroxynonenal (4-HNE), Malondialdehyde (MDA), and octanal 20. Octanal could activate the NLR family pyrin domain containing 3 (NLRP3) inflammasome and promote the secretion and release of IL-1β through the activation of olfactory receptor 2 (Olfr2), which is a member of the olfactory receptor family primarily distributed in sensory organs 21, 22. However, the relationship between ferroptosis in TIM-4^+^ smRTM promoting inflammatory responses and Olfr2 under EHS conditions requires further investigation.

In this study, we show that, under EHS conditions, heme oxygenase-1 (HMOX1) plays a central role in regulating ferroptosis of TIM-4⁺ smRTMs during the progression of RM. HMOX1-driven ferroptosis in TIM-4⁺ smRTMs increases octanal production and, through the transcription factor JunD, upregulates *Olfr2* expression, thereby activating NLRP3 inflammasomes and enhancing IL-1β production. By delineating this HMOX1-JunD-Olfr2-NLRP3 pathway, our work identifies TIM-4⁺ smRTM ferroptosis as a key amplifier of EHS-associated RM and suggests that targeting this axis may provide a basis for precise prevention and treatment of RM in patients with EHS.

## 2. Materials and methods

### 2.1 Study design

This study investigated the role of ferroptosis in TIM-4⁺ skeletal muscle-resident macrophages (smRTMs) during exertional heatstroke (EHS)-induced rhabdomyolysis (RM). A murine EHS model was established through forced treadmill running followed by heat exposure. The identity and distribution of smRTMs were analyzed using single-cell RNA sequencing, flow cytometry, and immunofluorescence. Ferroptosis in smRTMs was evaluated through transcriptomic profiling, iron/lipid peroxidation markers, and both pharmacological (ferrostatin-1) and genetic (Hmox1 CKO) inhibition. Functional relevance was assessed via clodronate liposome-mediated depletion and adoptive transfer of smRTMs. A co-culture system of TIM-4⁺ smRTMs with C2C12 myoblasts was used to examine cell-cell interactions. Finally, downstream mechanisms involving octanal production and Olfr2-NLRP3 inflammasome activation were explored. This integrated design combined *in vivo*, *ex vivo*, and *in vitro* approaches to define the pathological role of smRTM ferroptosis in muscle injury and repair.

### 2.2 Animal

All animal experiments were performed in compliance with the National Institutes of Health guidelines and were approved by the Animal Care and Use Committee of Nanfang Hospital (License No. IACUC-LAC-20230411-006). Male C57BL/6 wild-type (WT) mice (8-9 weeks old, sexually mature, specific pathogen-free) were obtained from the Southern Medical University Animal Center (Guangzhou, China). Four mice were housed per cage in a controlled facility with a 12 h light/12 h dark cycle, ambient temperature of 23 °C, and relative humidity of 55% ± 5%. Food and water were provided *ad libitum*.

Specific treatments were administered as follows:

- Clodronate liposomes (Clo Lip): 200 μL via tail vein injection; tested 48 h later (Ctrl Lip as control);
- Liproxstatin-1 (Lip-1): 10 mg/kg intraperitoneally (i.p.) 2 h prior to EHS induction;
- Deferoxamine (DFO): 100 mg/kg i.p. daily for 7 consecutive days;
- Vitamin E (VE): 500 mg/kg orally for 7 consecutive days;
- Zinc protoporphyrin (ZnPP): 10 mg/kg i.p., 24 h prior to experiment;
- Cobalt protoporphyrin (CoPP): 5 mg/kg i.p., 24 h prior;
- Raleukin, Anti-IL-6, and Infliximab: 30 mg/kg Raleukin, 5 mg/kg Anti-IL-6, and 10 mg/kg Infliximab i.p. for 2 consecutive days prior to the experiment;
- Citral: 10 mg/kg i.p., 24 h before the experiment.

To obtain macrophage-specific *Hmox1* knockout mice, B6.129P2-*Lyz2^tm1(cre)Ifo^*/J (004781, Jackson lab) were crossed with C57BL/6JCya-*Hmox1^em1flox^*/Cya (S-CKO-02911, Cyagen Biosciences), and the offspring were designated *Lyz2crexHmox1^flox/flox^*. *Nlrp3* knockout mice and *Caspase-1* knockout mice were purchased from Jackson lab (021302, B6.129S6-*Nlrp3^tm1Bhk^*/J; 016621, B6N.129S2-*Casp1^tm1Flv^*/J).

### 2.3 Experimental model

To establish a murine model of exertional heatstroke (EHS), male C57BL/6 mice were acclimatized to running wheels and allowed 2 days of rest with free access to exercise before the experiment. On the test day, animals were fasted for 1 h, and rectal temperature (Tc) was monitored. Mice were then placed in an artificial climate chamber maintained at 39.5 °C and 65% relative humidity (RH), where they ran on a motorized wheel at a constant speed of 15 rpm/min. EHS onset was defined as either a Tc reaching 43 °C or the appearance of exhaustion signs (inability to keep pace or righting reflex loss). At this point, mice were removed from the chamber, weighed, and transferred to a recovery environment (25 °C, *ad libitum* access to food and water). Tc was monitored for 24 h post-exposure. A classical heatstroke (CHS) model was induced under the same environmental conditions without exercise, using the same Tc or exhaustion criteria for termination. Two control groups were used: the sham heat rest (SHR) group remained in home cages (25 °C, 55% RH), while the sham heat exercise (SHE) group underwent exercise in a chamber at 27 °C and 55% RH for 1 h without heat exposure. All mice were weighed before and after experiments, and baseline Tc was measured twice daily for 2 weeks prior.

To simulate EHS-like stress *in vitro*, C2C12 myoblasts or smRTM cells were treated with 5 μM ionomycin (io) in DMEM to induce calcium overload. Cells were then subjected to heat stress (hs) at 43 °C in a humidified incubator with 5% CO₂ for 2 h, followed by recovery at 37 °C for 0, 6, or 12 h, as indicated. This protocol effectively mimics the combined effects of hyperthermia and intracellular calcium dysregulation observed in the EHS setting.

### 2.4 Primary cell isolation, flow cytometry, and culture

Mice were euthanized, and hindlimb muscles were excised, minced, and digested in 0.1% type II collagenase (V900892-1G, Sigma) at 37 °C for 30-60 min. After filtration (70 μm) and centrifugation (1500 rpm, 10 min), cells were resuspended in 30% Percoll, overlaid on 70% Percoll, and centrifuged at 1000 g for 30 min. The interphase was collected, washed, and stained with PerCP-Cy5.5 anti-CD45, APC anti-CD11b, APC/FITC anti-F4/80, and PE anti-CD64 antibodies. For TIM-4⁺ macrophage sorting, PE anti-TIM-4 was included. Flow cytometry was used to isolate CD45⁺CD11b⁺F4/80⁺CD64⁺ and TIM-4⁺ macrophages (BD FACSAria III).

Sorted cells were placed in complete DMEM (10% FBS and 1% penicillin-streptomycin) and supplemented with 20 ng/mL recombinant mouse M-CSF (PeproTech) to preserve RTM viability *ex vivo* 23. Where indicated, cells were pretreated with the following inhibitors: Necrostatin-1 (Nec-1, 50 μM, 2 h), 3-methyladenine (3-MA, 100 μM, 2 h), benzyloxycarbonyl-Val-Ala-Asp-fluoromethylketone (z-VAD-FMK, 10 μM, 2 h), Disulfiram (30 μM, 2 h), Ferrostatin-1 (Fer-1, 2 μM, 2 h), Liproxstatin-1 (Lip-1, 200 nM, 2 h), ZnPP (5 μM, 12 h), CoPP (5 μM, 12 h), and citral (10 μM, 2 h) prior to stress exposure.

### 2.5 Co-culture

Following the methodology described in a published study, we designed the co-culture system 24. The C2C12 cell line (1 × 10^5^ cells/mL) were first seeded into 24-well culture plates. Next, 0.2 × 10^5^ smRTMs were cultured on Transwell inserts with a 0.4 μm pore size. Myoblasts in the lower chamber were co-cultured before heatstroke treatment was initiated. Supernatant or cells were subsequently collected for analysis.

### 2.6 CCK8

The CCK-8 assay (Cell Counting Kit-8, Beyotime, Shanghai) was employed to evaluate cell viability in accordance with the manufacturer's instructions. Following the indicated treatments, cells grown in 96-well or 24-well plates were incubated with CCK-8 reagent at 37 °C for 2 h. The signal was then recorded at 450 nm using a microplate reader. After correcting for background, results were normalized to control wells to obtain relative viability.

### 2.7 Assay of ferrous ion

Tissue Fe²⁺ concentrations were measured using a commercial iron assay kit (I291, Dojindo, Japan). Tissues were weighed, homogenized in assay buffer, and centrifuged at 12,000 g for 10 min at 4 °C. Supernatants were mixed with chromogenic reagents, incubated at room temperature, and absorbance was read at 593 nm. Intracellular Fe²⁺ levels were evaluated using FerroOrange (F374, Dojindo) staining for 30 min at 37 °C and imaged with confocal fluorescence microscopy (Zeiss LSM980). All values were normalized to total protein content (BCA assay).

### 2.8 Measurement of malondialdehyde (MDA)

MDA levels in tissues and cells were measured using commercial kits (A106, Jiancheng, China; M496, Dojindo, Japan). After homogenization in MDA lysis buffer, samples were centrifuged and the resulting supernatants were used for the reaction. Thiobarbituric acid (TBA) was added and the mixtures were heated at 95 °C for 40 min; after cooling to room temperature, absorbance was measured at 532 nm. MDA content was calculated using standard curves and normalized to total protein concentration.

### 2.9 Measurement of Liperfluo

Lipid peroxidation was evaluated using the fluorescent probe Liperfluo (L248, Dojindo, Japan) according to the manufacturer's protocol. Treated cells were labeled with 1 μM Liperfluo at 37 °C for 30 min, followed by three PBS washes, and were promptly analyzed using flow cytometry on a BD FACSVerse instrument. Fluorescence intensity was quantified to reflect the degree of lipid ROS accumulation.

### 2.10 ELISA

Mouse plasma or cell culture supernatants were collected and analyzed using commercial ELISA kits. Interleukin-1β (IL-1β) and 4-Hydroxynonenal (4-HNE) levels were measured using kits CSB-E08054m and CSB-E13412m (CUSABIO, Wuhan, China), respectively. Myoglobin (Mb) concentrations were assessed using the CSB-E08050m kit (CUSABIO), following the manufacturer's protocols.

### 2.11 Creatine kinase (CK)

Under anesthesia, blood was drawn from mice via cardiac puncture. Plasma was isolated by centrifugation and analyzed for creatine kinase (CK) levels using an automated biochemical analyzer (BS-330E, Mindray, China) according to the manufacturer's instructions.

### 2.12 Flow cytometric analysis

Single cells isolated from mice tissues were labeled with the following antibodies: APC/FITC anti-F4/80 (ab105080, abcam and 12108, Biolegend), APC anti-CD11b (ab25482, abcam), PerCP/Cyanine5.5 anti-CD45(103132, Biolegend), APC anti-IL-1 beta (Pro-form) (17-7114-80, Invitrogen), PE anti-CD64 (139303, Biolegend), Alexa Fluor 647 anti-HMOX1 (ab237268, abcam), and PE anti-TIM-4 (12-5866-82, Invitrogen).

### 2.13 Plasmids and viruses

The following vectors were synthesized by Tsingke Biotechnology: *sh-Hmox1* (ACAGTGGCAGTGGGAATTTAT), *sh-Jund* (GTTCGCCGAAGGCTTCGTCAA), and *sh-Olfr2* (CACTGTTACGATTCCTAAGAT). A blank vector supplied by the same company was employed as a control. The resulting vector was transfected into 293T cells along with helper plasmids psPAX2 and pMD2.G to generate a viral solution. The viral solution was subsequently centrifuged and filtered before infecting the target cells.

### 2.14 Chromatin immunoprecipitation (ChIP) assay

Chromatin immunoprecipitation was performed using the SimpleChIP® Enzymatic Chromatin IP Kit (9003S, Cell Signaling Technology) according to the manufacturer's protocol. Briefly, cells were cross-linked, lysed, and sonicated to obtain sheared chromatin fragments. Immunoprecipitation was carried out using a JunD-specific antibody (PA1-834, Invitrogen) at a volume of 3.0-9.1 μg per reaction. Immunoprecipitated DNA was purified and subjected to quantitative PCR analysis to assess promoter binding activity.

### 2.15 Dual-luciferase reporter assay

The wild-type (WT) and mutant *Olfr2* promoter fragments (-2000 to +100 bp relative to the transcription start site) were cloned into the pEZX-FR03-Basic luciferase reporter vector (Genecopoeia). The mutant construct carried point mutations in the predicted Jund binding motif, as shown in Fig. 7C. HEK-293T cells were transfected with 1 μg/well of either WT or mutant* Olfr2* promoter plasmid using Lipofectamine 3000 (Thermo Fisher). In selected experiments, a *Jund* overexpression plasmid (pcDNA3.1*-Jund*) was co-transfected to assess transcriptional activation.

After 24 h of culture, cells were lysed and analyzed using the Luc-Pair™ Duo-Luciferase Assay Kit (LF004, Genecopoeia) following the manufacturer's instructions. Firefly and Renilla luciferase activities were measured using a Varioskan LUX multimode reader (Thermo Fisher), and relative luciferase activity was calculated after normalization to Renilla luciferase.

### 2.16 Tyramide signal amplification (TSA)-based immunofluorescent multiplex

Tissue samples, fixed and embedded, underwent immunofluorescence analysis. Briefly, slides were prepared by deparaffinization, antigen retrieval, and blocking. Antibodies (F4/80: GB11027, Service Bio and TIMD4:12008-1-AP, Proteintech) against specific markers were applied for staining. For cell slides, after fixation and peroxidase elimination, sequential co-staining with antibodies was performed. Slides were then incubated with a labeled antibody (NLRP3: ab4207, abcam and TGN38: ab16059, abcam) and processed using the TSA indirect kit (Jilin Histova) as per instructions. Confocal images were acquired on an Olympus microscope (Tokyo, Japan) and analyzed with ZEN Microscopy Software.

### 2.17 Single-cell RNA sequencing

Single-cell RNA sequencing was performed on skeletal muscle-resident tissue macrophages (smRTMs) isolated at different time points after EHS induction, including sham heat-rested controls (SHR), immediately after EHS (0 h), and 6 h post-recovery. Hindlimb skeletal muscles were harvested, enzymatically digested, and processed into single-cell suspensions. Macrophages were enriched via flow cytometry using surface markers CD45⁺, CD11b⁺, F4/80⁺, and CD64⁺. Cell viability, determined using trypan blue exclusion, exceeded 90% in all samples.

Cells were resuspended in PBS (HyClone) at 2 × 10⁵ cells/mL and loaded into microwell chips via the Singleron Matrix® Single Cell Processing System. Barcoding beads captured mRNA from individual cells, followed by reverse transcription and cDNA amplification. The resulting cDNA was fragmented and ligated with sequencing adapters. scRNA-seq libraries were prepared using the GEXSCOPE® Single Cell RNA Library Kit (Singleron) and sequenced on the Illumina NovaSeq 6000 platform (150 bp paired-end reads) 25. Raw sequencing data were processed with CeleScope v1.1.4 (Singleron) to generate gene expression matrices. Downstream analysis was performed in Seurat v3.1.2, including quality control, normalization (NormalizeData), scaling (ScaleData), dimensionality reduction, and clustering. Differential expression analysis was corrected using the Bonferroni method, with adjusted *P* < 0.05 considered significant.

### 2.18 Quantitative Real-time PCR (qPCR)

Total RNA was extracted from cells using RNA extraction reagent (G3326-15, Servicebio) in accordance with the manufacturer's instructions. The mRNA was reverse transcribed into cDNA using SweScript All-in-One RT SuperMix (G3337-100, Servicebio). Quantification of mRNA expression levels was carried out using 2×Universal Blue SYBR Green qPCR Master Mix (G3326-15, Servicebio), followed by normalization to the mRNA level of *Gapdh*. Specific primer sequences are provided in the Table 1.

### 2.19 Western blotting

Tissues and cells were lysed using total protein lysis buffer (P0013, Beyotime), followed by quantification of protein concentrations through a BCA protein assay kit (DQ111-01, TransGen, Beijing, China). For Western blotting, the membranes were probed with primary antibodies against the following targets: pro-Caspase-1+p10+p12 (ab179515, abcam), OR2H2 (ES14379, ELK Biotechnology), JunD (PA1-834, Invitrogen), and Tubulin (66031-1, Proteintech). Membranes were then incubated with peroxidase-conjugated secondary antibodies (1:5000), and the resulting signals were developed by enhanced chemiluminescence.

### 2.20 Statistical analysis

Data are reported as mean ± SEM, with individual mouse values overlaid on all graphs to show their distribution. Statistical significance was determined in GraphPad Prism 10 by one-way ANOVA with Tukey's post hoc test. Kaplan-Meier survival analyses were evaluated using the log-rank (Mantel-Cox) test. Differences were considered significant when *P* < 0.05.

## 3. Results

### 3.1 smRTM induced ferroptosis promotes RM progression

**Figure 1:**
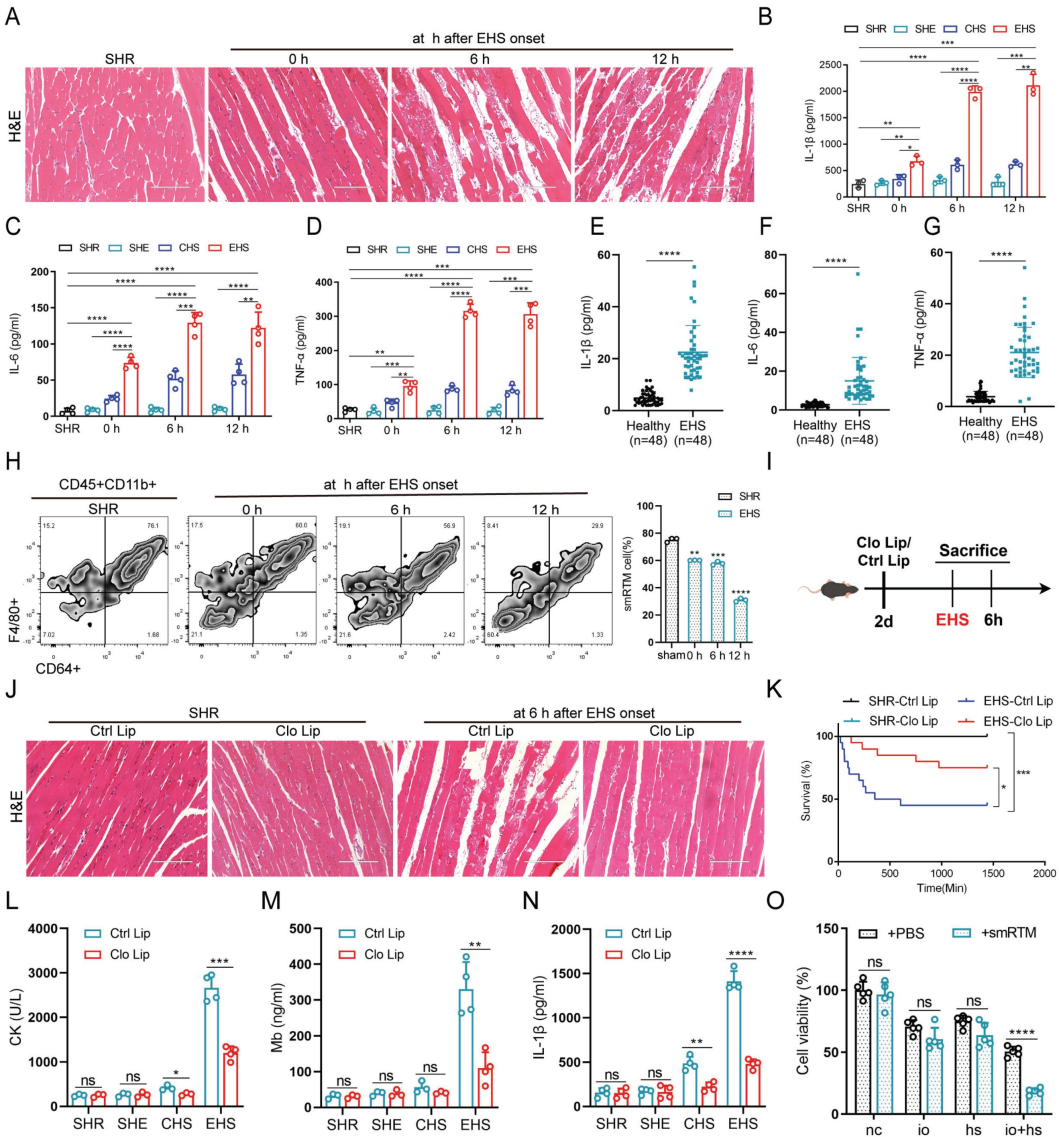
** smRTM contributes to skeletal muscle injury in RM during EHS (A)** Representative histological images of skeletal muscle sections stained with Hematoxylin and Eosin (HE) from EHS mice. **(B-D)** Plasma levels of IL-1β, IL-6, and TNF-α were measured in EHS mice (n=3-4).** (E-G)** The levels of IL-1β, IL-6, and TNF-α in the serum of EHS patients and healthy individuals were assayed. **(H)** Flow cytometry assessed smRTM frequency in EHS mice at 0 h, 6 h, and 12 h post-recovery (n=3 mice/group). **(I)** Schematic illustration of smRTMs depletion achieved via Clodronate Liposomes (Clo Lip) compared with Control Liposomes (Ctrl Lip). **(J)** Representative histological images of skeletal muscle sections from EHS mice pretreated with Clo Lip or Ctrl Lip. **(K)** Survival curves of pretreatment mice (n = 20 mice/group). **(L-N)** In parallel, plasma creatine kinase (CK) levels, MB and IL-1β level were measured (n = 3-4 mice/group). **(O)** Cell viability was assessed in co-cultured C2C12 cell with smRTMs at 6 h post io+hs induction. Summary data are presented as mean ± SEM, and statistical significance was determined using the Student's t-test. (*P<0.05, **P<0.01, ***P<0.001, ****P<0.0001). Scale bar = 200 μm.

In the murine model, we noted reduced muscle fiber density, disorganized fiber arrangement, and pronounced swelling degeneration after EHS onset (Fig. 1A). Additionally, creatine kinase (CK) and myoglobin (Mb) levels significantly increased (Fig. S1A, B). Meanwhile, we observed elevated levels of inflammatory factors such as Interleukin-1β (IL-1β), Interleukin-6 (IL-6), and Tumor Necrosis Factor-α (TNF-α) in both animal models and EHS patients (Fig. 1B-G). To investigate smRTM's role in RM during EHS, we isolated smRTM cells using flow cytometry. Compared to SHR group, smRTM cell numbers significantly declined in EHS mice (Fig. 1H). Further investigating smRTM, we administered intraperitoneal Clodronate Liposomes (Clo Lip) to deplete muscle macrophages before EHS treatment (Fig. 1I and S1C). The Clo Lip group of EHS mice showed higher survival rates than the Control Liposomes (Ctrl Lip) Group, with reduced skeletal muscle injury and inflammatory factors (Fig. 1J-N and S1D, E). Moreover, in a co-culture arrangement, primary smRTMs were seeded in transwell inserts, and C2C12 cells were placed in a 24-well plate. Assessment revealed an increase in myoblast death when co-cultured with smRTMs (Fig. 1O). These results suggest that EHS alters the abundance and functional state of smRTMs, which may indirectly contribute to skeletal muscle injury.

**Figure 2:**
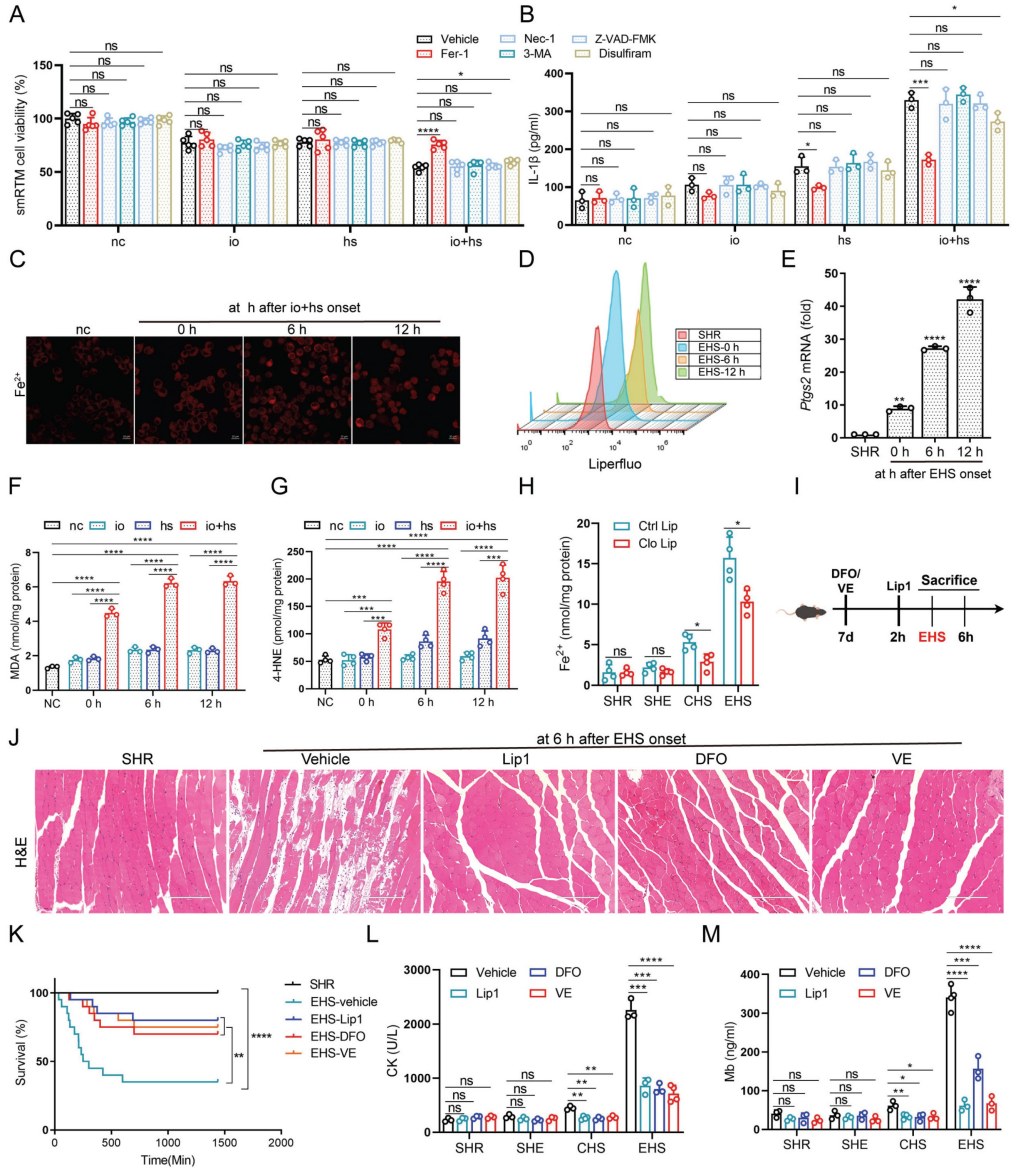
** The role of smRTM ferroptosis in promoting skeletal muscle injury in EHS mice (A-B)** After pretreatment with various inhibitors, smRTM cell viability and IL-1β level were assessed at 6 h post io+hs induction exposure (n=3-5).** (C)** Representative FerroOrange staining images in smRTM after io+hs exposure. **(D)** Flow cytometry was employed to analyze lipid peroxidation in smRTM (n = 3 mice/group).** (E)** Relative levels of *Ptgs2* were measured in smRTM from EHS mice (n = 3 mice/group). **(F-G)** Level of MDA and 4-HNE were assayed (n = 3-4).** (H)** The level of tissue ferrous ion at 6 h were assayed (n = 3-5 mice/group).** (I)** Schematic illustration depicting the modulation of ferroptosis using Liproxstatin-1(Lip1), Deferoxamine (DFO) and Vitamin E (VE)**. (J)** Representative histological images from EHS mice pretreated with Lip1, DFO and VE.** (K)** Survival curves of pretreated mice. (n=20 mice/group). **(L-M)** Detection of CK and Mb in plasma (n=3-4). All samples were collected at 6 h post-EHS onset. Summary data are presented as mean ± SEM, and statistical significance was determined using the Student's t-test. (*P<0.05, **P<0.01, ***P<0.001, ****P<0.0001). scale bar = 10 μm.

To explore heat-induced cytotoxicity mechanisms in smRTM, we assessed cell viability with various cell death inhibitors (Fig. 2A). Our findings indicated that ferroptosis inhibitor (Fer-1) significantly reduced heat-induced cell death, suggesting the implication of ferroptosis in this process. Additionally, Fer-1 markedly decreased IL-1β production under io+hs conditions (Fig. 2B). To confirm these findings, we isolated smRTM and labeled them with the intracellular iron ion fluorescence probe FerroOrange and the lipid peroxidation probe Liperfluo. We observed increased intracellular iron content and lipid peroxidation in these cells (Fig. 2C, D). The mRNA expression of prostaglandin-endoperoxide synthase 2 (*Ptgs2*), a universal marker of ferroptosis, showed the highest elevation following EHS (Fig. 2E). Additionally, levels of MDA and 4-HNE significantly increased under EHS conditions (Fig. 2F, G). However, depleting smRTM resulted in decreased tissue ferrous ion, MDA, and 4-HNE levels (Fig. 2H and S1F, G). Furthermore, pre-treatment of mice with ferroptosis inhibitors such as Vitamin E (VE), Liproxstatin-1 (Lip1), and Deferoxamine Mesylate (DFO) increased survival rates and mitigated skeletal muscle injury in RM following EHS (Fig. 2I-M).

To summarize, the results suggest that ferroptosis of smRTM significantly contributes to the promotion of skeletal muscle injury under EHS conditions.

### 3.2 Single-cell RNA sequencing revealed TIM-4^+^ smRTM subset sensitive to ferroptosis

**Figure 3:**
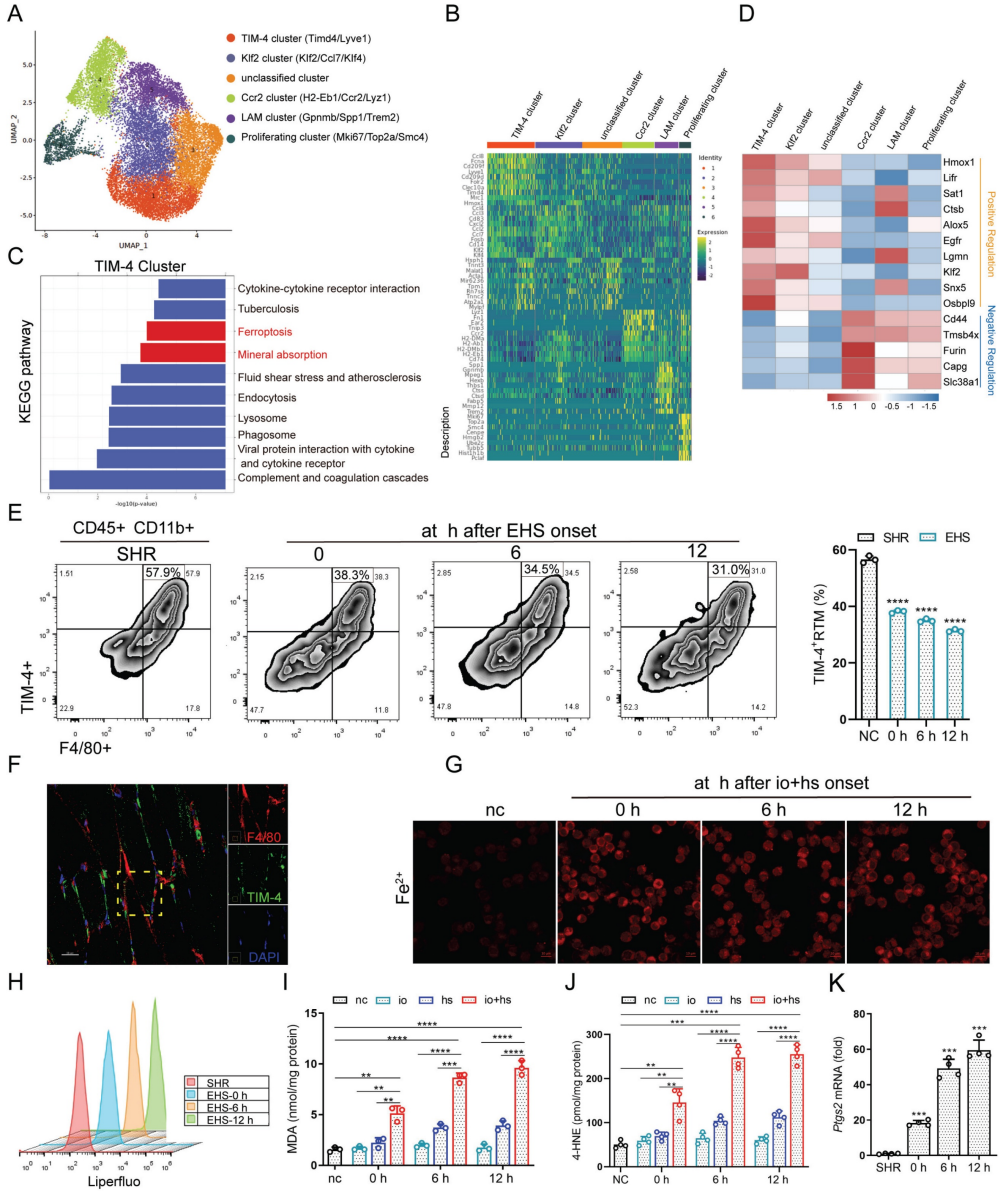
** TIM-4^+^ smRTM shows distinct susceptibility to ferroptosis (A)** UMAP visualization of macrophage subsets. **(B)** Marker genes used for cluster annotation.** (C)** KEGG pathway enrichment analysis of up-regulated differentially expressed genes (DEGs) identified ferroptosis-related genes highly expressed in TIM-4 cluster. **(D)** Heat maps illustrate the expression of genes related to iron ion metabolism, lipid metabolism, and oxidant metabolism in each cluster, based on single-cell RNA-seq. **(E)** Flow cytometry was used to monitor the frequency of TIM-4^+^ smRTM in EHS mice during recovery at 0 h, 6 h, and 12 h (n = 3 mice/group). **(F)** Representative immunofluorescence images of F4/80 (red), TIM-4 (green), and DAPI (green) in in skeletal muscle. **(G)** FerroOrange staining of TIM-4⁺ smRTMs following io+hs exposure. **(H)** Lipid peroxidation in TIM-4⁺ smRTMs detected by flow cytometry (n = 3).** (I-J)** MDA and 4-HNE levels in TIM-4⁺ smRTMs (n = 3-4).** (K)** *Ptgs2* mRNA expression in TIM-4⁺ smRTMs** (n = 4).** Summary data are presented as the mean ± SEM. Significance was calculated using the Student's t-test. (*P<0.05, **P<0.01, ***P<0.001, ****P<0.0001).

The RTM resident microenvironment evolves under the influence of skeletal muscle tissue, resulting in a highly heterogeneous RTM phenotype with diverse functions 13. To explore this, we isolated smRTM and conducted single-cell transcriptome sequencing analysis. Using Seurat analysis with Uniform Manifold Approximation and Projection (UMAP) for dimension reduction and automatic clustering, we identified six primary clusters (Cluster 1 to 6) (Fig. 3A). Based on previously described features and nomenclature in smRTM 26, as well as the analysis of differentially expressed genes (Fig. 3B and S2A, B), we distinguished five distinct cell types. Cluster 1 was characterized as T cell membrane protein 4-positive (TIM-4^+^) macrophages, Cluster 2 as Klf2^+^ macrophages, Cluster 4 as Ccr2^+^ macrophages, Cluster 5 as lipid-associated macrophages (LAM), and Cluster 6 indicated proliferating macrophages. We then performed KEGG pathway analysis on the differentially expressed genes of the annotated clusters. The results highlighted a significant association between TIM-4^+^ smRTM and ferroptosis pathways (Fig. 3C), while other macrophage subsets didn't show enrichment in ferroptosis-related pathways (Fig. S2C). Considering the functional roles of ferroptosis-related genes, we evaluated cluster susceptibility to ferroptosis. TIM-4⁺ smRTMs showed up-regulation of genes that positively regulate ferroptosis and down-regulation of anti-ferroptotic genes, indicating an increased ferroptosis-prone transcriptional state (Fig. 3D). In the Gene Ontology (GO) analysis, we observed pathways linked to cellular responses to oxidative stress and iron ion homeostasis enriched in the TIM-4^+^ cluster compared to others (Fig. S2D). Additionally, Gene Set Enrichment Analysis highlighted distinct functional roles within various subsets. The subset predicted to be sensitive to ferroptosis (TIM-4^+^ cluster) showed significant enrichment of genes related to cellular iron ion homeostasis (Fig. S2E).

Moreover, KEGG pathway analysis results reveal that, compared to the negative control group (NC), the differentially expressed genes in the EHS-conditioned recovery 0 h samples are enriched in the ferroptosis pathway in TIM-4^+^ cluster (Fig. S2F). Remarkably, ferroptosis pathway enrichment was already evident at recovery 0 h, indicating rapid transcriptional activation immediately after heat stress and suggesting an early susceptibility of TIM-4⁺ macrophages to ferroptotic stress. We subsequently validated the presence of TIM-4^+^ smRTM in skeletal muscle sections using multicolor fluorescence imaging and confirmed a decrease in their abundance in EHS mice through flow cytometry (Fig. 3E, F). Under *in vitro* EHS conditions, we observed an increase in intracellular iron content and the occurrence of lipid peroxidation in these cells (Fig. 3G, H). We also found that levels of MDA and 4-HNE increased compared to the NC group under* in vitro* EHS conditions (Fig. 3I, J). Furthermore, *Ptgs2* mRNA exhibited the highest increase after EHS (Fig. 3K). Moreover, TIM-4⁺ smRTMs exhibited markedly elevated lipid ROS levels, as evidenced by increased Liperfluo staining intensity and flow cytometric shifts, along with significantly higher MDA and 4-HNE content compared to TIM-4⁻ smRTMs under io+hs conditions (Fig. S3A-E). These findings imply that TIM-4⁺ smRTMs, with their enrichment of ferroptosis-related genes, exhibit higher susceptibility to ferroptosis induction. Together, these data underscore the detrimental role of TIM-4⁺ smRTMs in regulating skeletal muscle injury during EHS.

### 3.3 HMOX1 mediated ferroptosis contributes to rhabdomyolysis development following EHS

**Figure 4:**
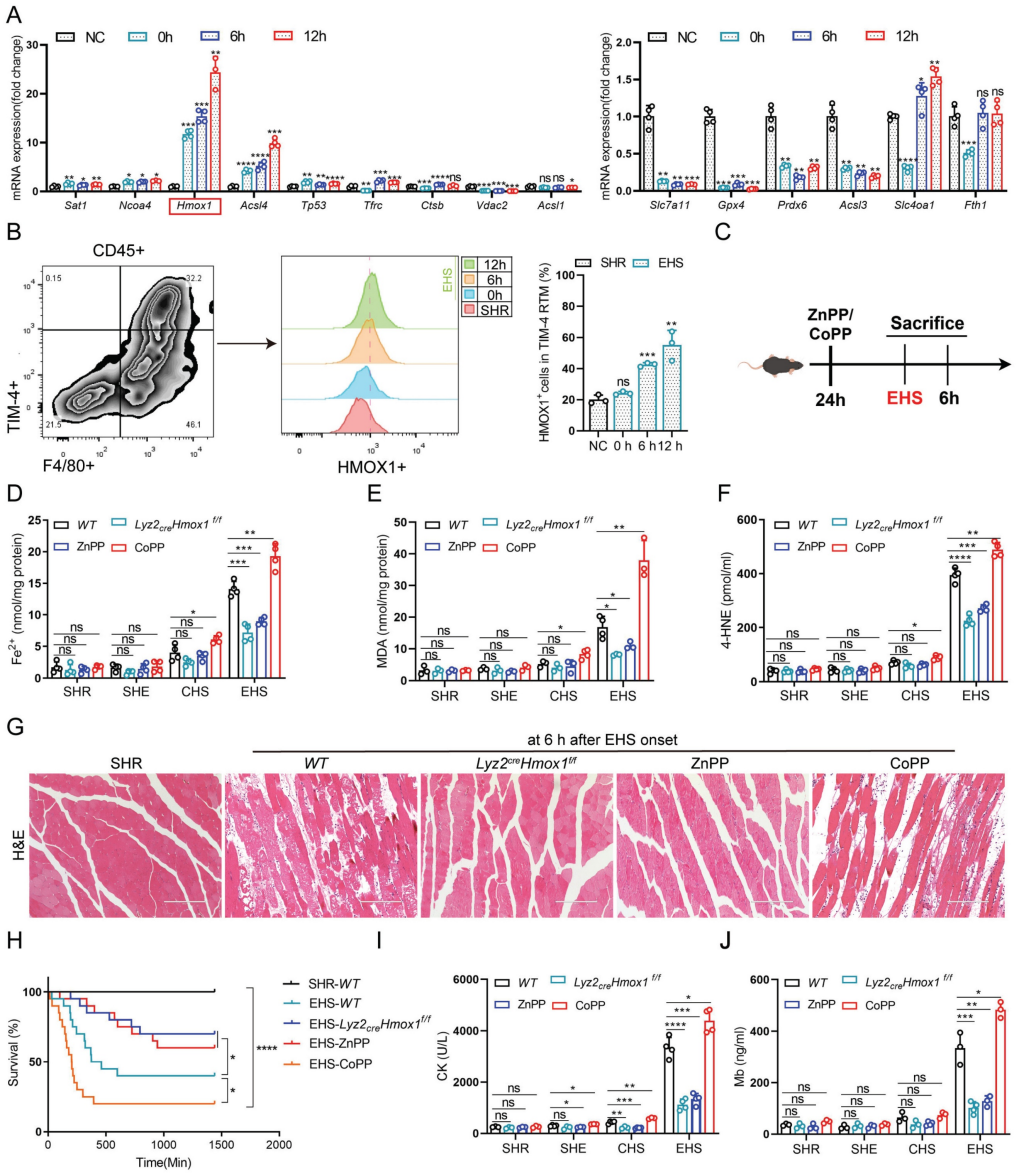
** Hmox1-mediated ferroptosis contributes to RM development following EHS (A)** Relative expression of ferroptosis-related genes in FACS-purified TIM-4⁺ smRTMs after io+hs induction (n = 3) **(B)** Flow cytometry detecting HMOX1⁺ TIM-4⁺ smRTMs in skeletal muscle (n = 3). **(C)** Schematic showing modulation of HMOX1 activity. **(D-F)** Levels of tissue ferrous ion **(D)**, MDA **(E)**, and 4-HNE **(F)** were assessed at 6 h (n = 3-4 mice/group). **(G)** HE staining of skeletal muscle sections following ZnPP, CoPP, or smRTM *Hmox1* knockout.** (H)** Survival curves of pretreated mice (n = 20 mice/group). **(I-J)** Plasma CK and Mb levels were detected in pretreated mice. All samples were collected at 6 h post EHS onset. Summary data are presented as mean ± SEM, and statistical significance was determined using the Student's t-test. (*P<0.05, **P<0.01, ***P<0.001, ****P<0.0001).

To further investigate ferroptosis-related gene expression, we isolated TIM-4⁺ smRTM cells and conducted qPCR analysis. Among the tested genes, *Hmox1* exhibited the most pronounced upregulation in the EHS group (Fig. 4A). Moreover, flow cytometry analysis revealed a higher proportion of HMOX1⁺ TIM-4⁺ smRTMs at multiple recovery time points post-EHS (Fig. 4B). Based on these observations, we selected *Hmox1* for subsequent mechanistic studies.

To comprehensively confirm the role of HMOX1 in TIM-4^+^ smRTM ferroptosis, we assessed whether changes in *Hmox1* expression influenced ferroptosis-related markers in mouse smRTM (Fig. 4C). Inhibiting HMOX1 with zinc protoporphyrin (ZnPP) reduced ferroptosis markers, including free iron, MDA, and 4-HNE in EHS mice (Fig. 4D-F). Conversely, activating HMOX1 with cobalt protoporphyrin reversed these outcomes (CoPP). To create a smRTM *Hmox1* knockout model, we bred *Hmox1^flox/flox^* mice with* Lyz2-cre-tdTomato* mice, resulting in mice with smRTM* Hmox1* knockout, designated as *Lyz2_crex_Hmox1^f/f^*. Upon EHS challenge, this model exhibited reduced ferroptosis marker levels, including free iron, MDA, and 4-HNE (Fig. 4D-F), suggesting a protective effect of *Hmox1* deletion in smRTMs against ferroptosis. *In vitro* EHS model, we similarly observed that inhibiting HMOX1 with ZnPP or inducing *Hmox1* knockout in smRTM cells could effectively suppress cell death and ferroptosis markers, including ferrous ion, Liperfluo, MDA, and 4-HNE. Conversely, the results were reversed upon activation of HMOX1 through CoPP treatment (Fig. S4A-E). Furthermore, suppression of HMOX1 using ZnPP or *Hmox1* knockout in smRTM cells resulted in reduced skeletal muscle injury and enhanced mouse survival rates. Conversely, the HMOX1 agonist CoPP produced the opposite outcome (Fig. 4G-J). In a co-culture model of TIM-4^+^ smRTM and C2C12 cells, we provided additional evidence for this phenomenon (Fig. S4F). Collectively, these findings indicate that HMOX1-mediated ferroptosis in TIM-4⁺ smRTMs plays a pivotal role in driving skeletal muscle injury during EHS.

### 3.4 Ferroptosis dependent on HMOX1 in TIM-4^+^ smRTM promotes the generation of IL-1β

**Figure 5:**
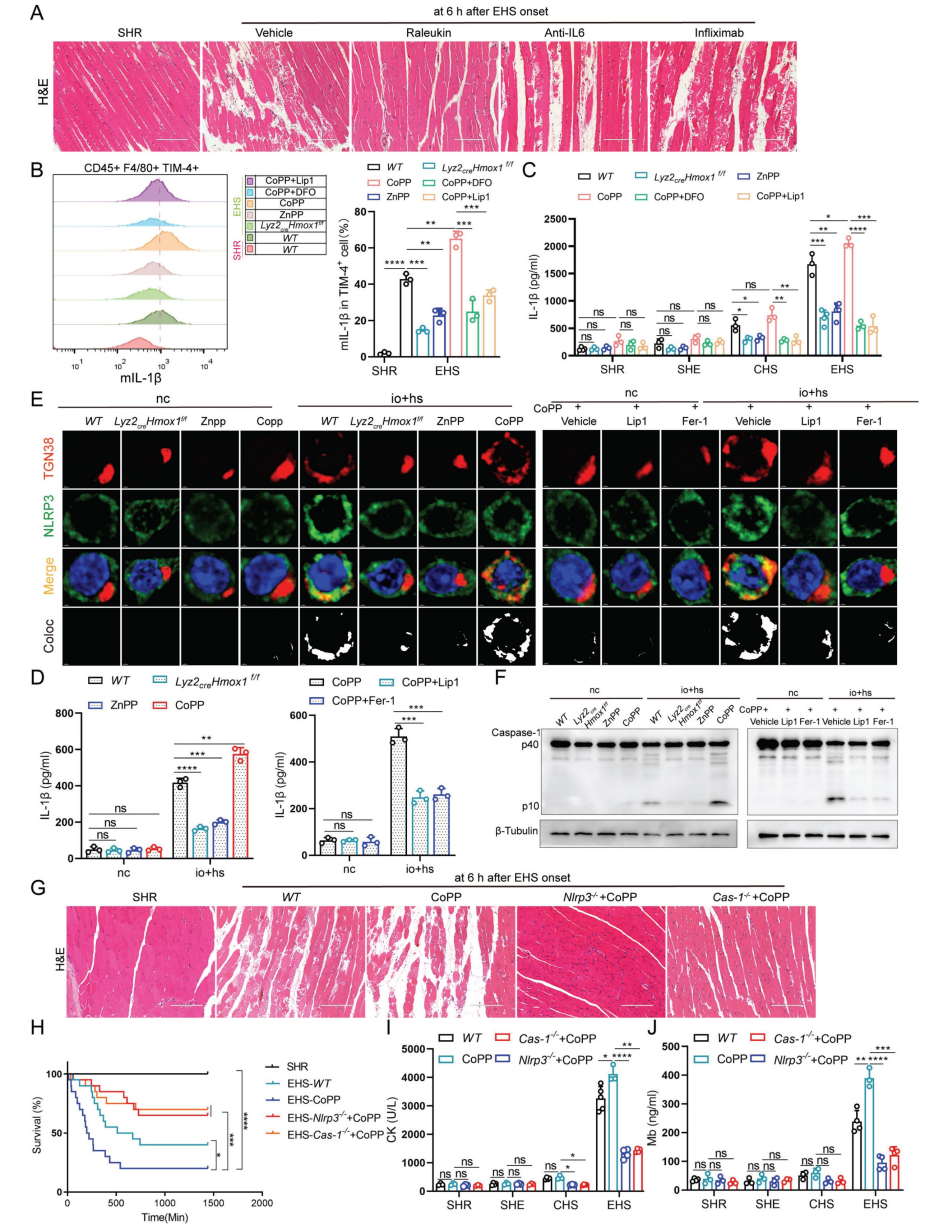
**HMOX1-dependent TIM-4^+^ smRTM ferroptosis promotes NLRP3 inflammasome activation (A)** HE-staining of skeletal muscle sections is displayed for mice pretreated with Raleukin, Anti-IL-6, and Infliximab, followed by EHS.** (B)** Flow cytometry was employed to detect mIL-1β expression in F4/80^+^/TIM-4^+^ cells in skeletal muscle tissues, and statistical results are provided (n = 3 mice/group).** (C)** Plasma IL-1β content were detected (n = 3-5 mice/group). **(D)** Detection of IL-1β levels in cell supernatants (n = 3). **(E)** Representative images of immunofluorescence staining for TGN38 (red), NLRP3 (green), and DAPI (blue) in *WT* and TIM-4^+^ smRTM from *Lyz2_crex_Hmox1^f/f^* pretreated with Znpp, Copp, *Hmox1* knockout, Copp+Lip1, or Copp+Fer-1 at 6 h after io+hs induction exposure. **(F)** Western Blotting analysis was performed to assess Caspase-1 expression. **(G)** Representative HE-staining of skeletal muscle sections of mice pretreated with CoPP of WT mice, *Nlrp3^-/-^* mice or* Cas-1^-/-^* mice, followed by EHS.** (H)** Survival curves of pretreated mice (n = 20 mice/group). **(I-J)** Detection of CK and Mb in plasma of pretreated mice (n = 3-5 mice/group). All samples were collected at 6 h after EHS onset. Summary data are presented as the mean ± SEM. Significance was calculated using the Student's t-test. (*P<0.05, **P<0.01, ***P<0.001, ****P<0.0001).

Previous studies have noted a significant rise in inflammatory factors levels in both EHS patients and animal models (Fig. 1B-G). Thus, we administered neutralizing antibodies targeting these factors individually to EHS animal models (Fig. S5A). Interestingly, only the IL-1β neutralizing antibody effectively suppressed RM progression under EHS conditions (Fig. 5A and S5B-D). Furthermore, our studies showed a close association between IL-1β concentrations and smRTM ferroptosis (Fig. 2B). Subsequently, suppressing HMOX1 with ZnPP or* Hmox1* knockout in smRTM cells reduced mIL-1β-positive cells under EHS conditions, while CoPP treatment increased them. CoPP combined with DFO or Lip1 effectively countered CoPP-induced effects (Fig. 5B). Knocking out or inhibiting HMOX1 reduced plasma IL-1β secretion, while CoPP administration increased it in EHS mice; DFO or Lip1 reversed this (Fig. 5C). We further confirmed these findings under* in vitro* io+hs conditions (Fig. 5D and S5E).

The study suggests that NLRP3 inflammasome activation triggers caspase-1 cleavage, leading to IL-1β release 27. Our analysis revealed significant enrichment of NLRP3 inflammasome-related genes in TIM-4^+^ smRTM (Fig. S6A, B). Compared to the NC group, EHS showed increased expression of genes associated with the NLRP3 inflammasome pathway (Fig. S6C, D). To delve deeper into EHS's impact on NLRP3 activation, we stained io+hs treated TIM-4^+^ smRTM cells for NLRP3 and trans-Golgi network marker TGN38, observing increased co-localization (Fig. S7A). Western blotting revealed heightened cleaved caspase-1 in the EHS group (Fig. S7B), while flow cytometry showed increased HMOX1 and Caspase-1-positive TIM-4^+^ smRTM cells under io+hs conditions (Fig. S7C). Additionally, we investigated NLRP3 inflammasome's role in EHS-induced skeletal muscle injury using *Nlrp3* and* Caspase-1* knockout models. The findings indicated that the knockout of *Nlrp3* or *Caspase-1* resulted in resulted in decreased skeletal muscle injury and improved the survival rate of mice in RM during EHS (Fig. S8A-D), and reduced levels of IL-1β in the plasma of EHS mice (Fig. S8E).

Moreover, our results found that the knockout or inhibition of HMOX1 reduced the co-localization of NLRP3 and TGN38, while HMOX1 activation with CoPP increased their co-localization. Co-treatment with Fer-1 or Lip1 reversed the effects induced by CoPP (Fig. 5E and S8F). Similarly, we further validated this phenomenon by assessing the levels of cleaved Caspase-1 using both western blotting and flow cytometry (Fig. 5F and S8G, H). Subsequently, to examine the role of NLRP3 inflammasome activation in EHS-induced skeletal muscle injury, we treated EHS mice with CoPP (Fig. S8I) and observed an exacerbation of injury and a decrease in mouse survival. Conversely, knockout of *Nlrp3* or *Caspase-1* reversed this effect (Fig. 5G-J). These findings suggest that HMOX1-dependent TIM-4^+^ smRTM ferroptosis, through NLRP3 activation, mediates the production of IL-1β and contributes to RM progression.

### 3.5 HMOX1 dependent ferroptosis promotes IL-1β production via olfactory receptor 2

The mechanisms linking ferroptosis to NLRP3 activation during EHS remain unclear. Notably, ferroptotic stress has been associated with increased production of lipid aldehydes, including octanal 20. Octanal can activate NLRP3 inflammasome through olfactory receptor 2 (Olfr2) and promote the secretion and release of IL-1β 21. Next, we investigate whether the levels of octanal and *Olfr2* expression are affected under EHS conditions. We used liquid chromatography to measure octanal levels in mouse plasma, and the results showed an increase in octanal content in the EHS group compared to NC group (Fig. S9A). Similarly, qPCR and western blot results revealed a significant upregulation of *Olfr2* expression in TIM-4^+^ smRTM under* in vitro* EHS conditions (Fig. S9B, C). To compare the effects of different lipid peroxidation-derived aldehydes on inflammasome activation, TIM-4⁺ smRTMs were treated with octanal, MDA-BSA, or 4-HNE. Among these aldehydes, octanal induced a markedly stronger increase in caspase-1 activation and IL-1β secretion under io + hs conditions, whereas MDA-BSA and 4-HNE showed minimal or modest effects (Fig. S9D-F).

**Figure 6:**
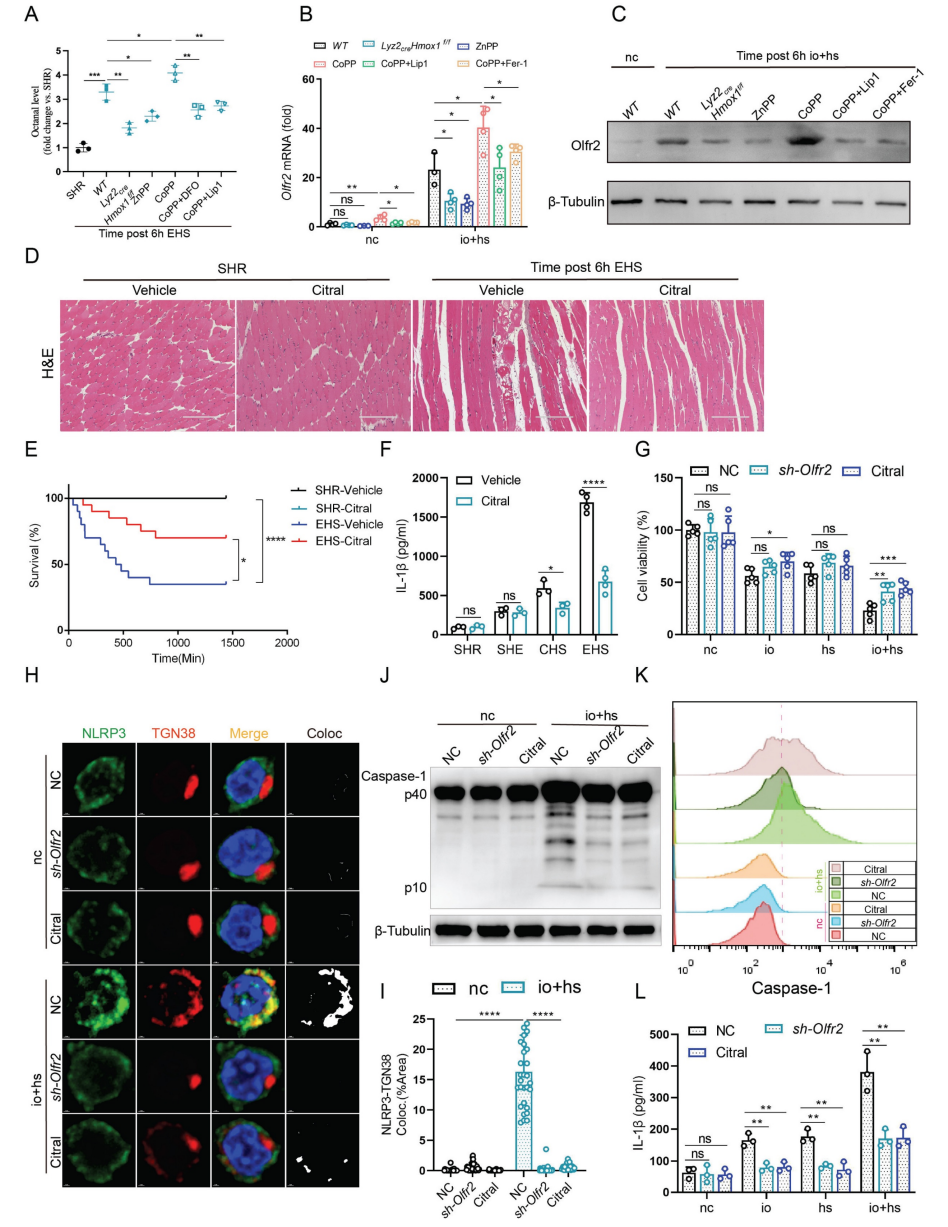
** HMOX1-dependent ferroptosis in TIM-4^+^ smRTM promotes NLRP3 activation via olfactory receptor 2 (A)** Octanal levels in the plasma of *Lyz2_crex_Hmox1^f/f^* mice or mice pretreated with ZnPP, CoPP, *Hmox1* knockout, CoPP+Lip1, or CoPP+Fer-1 were measured at 6 h after EHS exposure (n = 3 mice/group). **(B-C)** The expression of Olfr2 in TIM-4^+^ smRTM pretreated Zn, CoPP, CoPP+Lip1, CoPP+Fer-1 were assayed by qPCR **(B)** and Western bloting **(C)** at 6 h following io+hs exposure. **(D)** Representative HE-staining of skeletal muscle sections of mice pretreated DMSO or Citral, followed by EHS. **(E)** Survival curves were generated for mice (n = 20 mice/group). **(F)** Plasma IL-1β content were detected. (n = 3-5 mice/group).** (G)** Cell viability was assessed in TIM-4^+^ smRTM pretreated with *sh-Olfr2* or Citral at 6 h after io+hs induction exposure.** (H-I)** Representative images of immunofluorescence in TIM-4^+^ smRTM and statistical analysis of co-localization of NLRP3 and TGN38. **(J-K)** Western Blotting **(J)** and flow cytometry** (K)** were employed to analyze Caspase-1 expression in TIM-4^+^ smRTM. **(L)** Detection of IL-1β levels in cell supernatants (n = 3). Summary data are presented as the mean ± SEM. Significance was calculated using the Student's t-test. (*P < 0.05, **P < 0.01, ***P < 0.001, ****P < 0.0001).

Further investigations revealed that inhibition of HMOX1 with ZnPP or* Hmox1* knockout in smRTM reduced the levels of octanal in plasma and suppressed* Olfr2* expression in TIM-4^+^ smRTM. Conversely, the results were reversed with the activation of HMOX1 by injection of CoPP. Additionally, co-treatment with ferroptosis inhibitors (DFO, Lip1, and Fer-1) reversed the effects induced by CoPP (Fig. 6A-C). Next, to explore the impact of Olfr2 on EHS-induced skeletal muscle injury, we administered Citral (an Olfr2 inhibitor) to EHS mice and observed reduced mortality and skeletal muscle injury (Fig. 6D, E and S9G-I). Inhibition of Olfr2 with Citral also led to a reduction in plasma IL-1β secretion in EHS mice (Fig. 6F). Additionally, in an* in vitro* co-culture model of TIM-4^+^ smRTM and myoblasts, we noticed that inhibiting or downregulating *Olfr2* expression could suppress the mortality of C2C12 cells under EHS conditions (Fig. 6G). Further investigations revealed that, under* in vitro* EHS conditions, inhibition or downregulation of *Olfr2* expression could reduce the co-localization of NLRP3 and TGN38 (Fig. 6H, I), formation of Caspase-1 mature bodies (Fig. 6J, K), and IL-1β secretion (Fig. 6L).

### 3.6 JunD regulates the transcription of Olfr2

**Figure 7:**
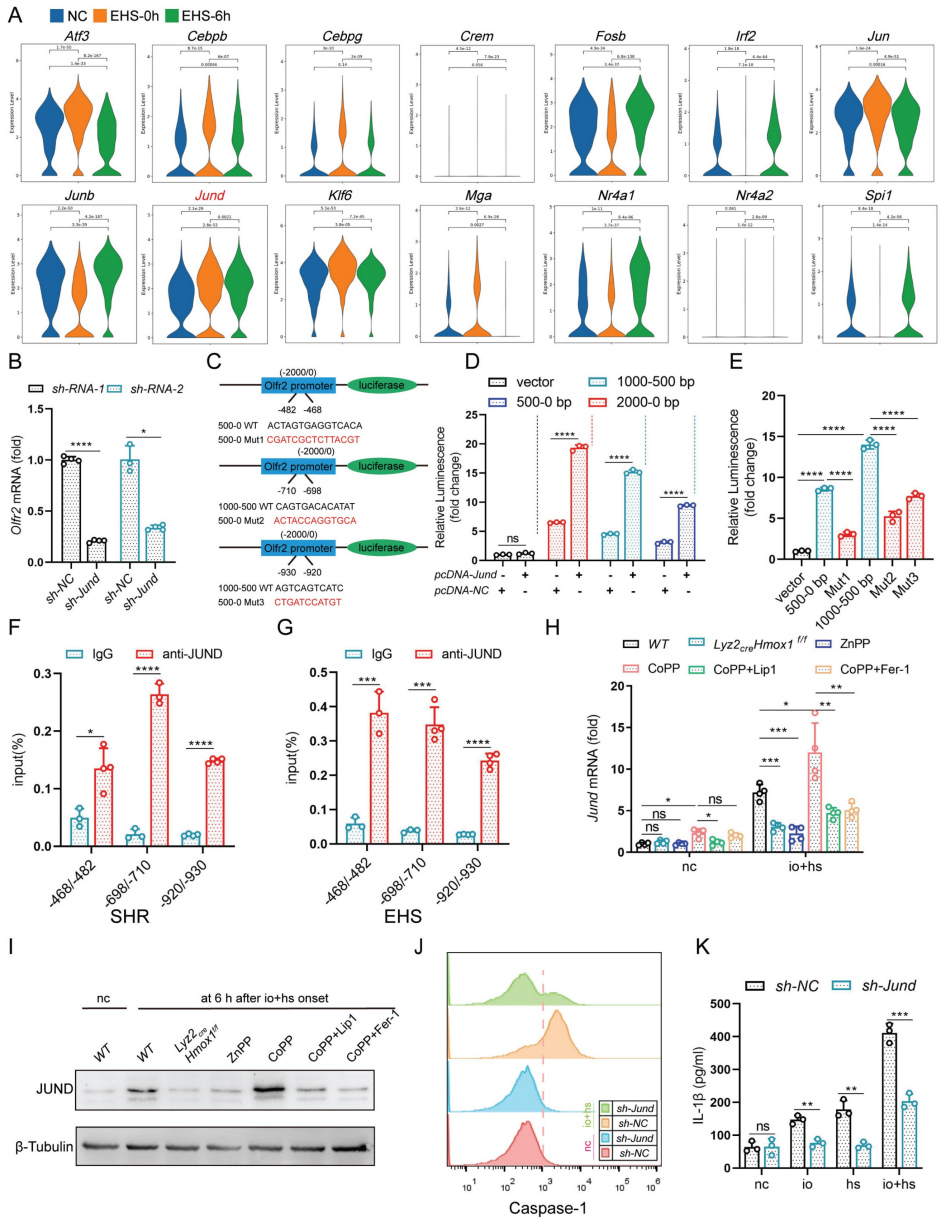
** JunD regulates *Olfr2* transcription and induces NLRP3 activation (A)** Violin graph showing the expression of* Atf3*, *Cebpb*, *Cebpg*, *Crem*, *Fosb*, *Irf2*, *Jun*, *Junb*, *Jund*, *Klf6*, *Mga*, *Nr4a1*, *Nr4a2*, and* Spi1* under EHS conditions compared to the NC group. **(B)** qPCR analysis of *Olfr2* expression after knocking down* Jund* (*sh-Jund*)(n=3-4).** (C)** Schematic diagram of site-directed mutagenesis of -482~-468, -710~-698, -930~-920 sites in the *Olfr2* promoter region.** (D-E)** Dual-luciferase experiments in HEK-293T cells transfected with various truncated regions **(D)** or various mutational regions (n=3)** (E)** of the *Olfr2* promoter. **(F-G)** ChIP-PCR analysis of JunD binding to sites -468~-482, -698~-710, -920~-930 in the *Olfr2* promoter region (left) and fold change in signals (right) after io+hs treatment(n=3-4). **(H-I)** The expression of *Jund* in TIM-4^+^ smRTM pretreated ZnPP, CoPP, CoPP+Lip1, CoPP+Fer-1 were assayed by qPCR **(H)** and Western bloting** (I)** at 6 h following io+hs exposure (n=3-4). **(J)** Caspase-1 expression in TIM-4^+^ smRTM were detected by flow cytometry. **(K)** Detection of IL-1β content in the supernatant(n=3). Summary data, presented as mean ± SEM, underwent statistical analysis using the Student's t-test. (*P<0.05, **P<0.01, ***P<0.001, ****P<0.0001).

We then investigated the transcriptional regulation of *Olfr2* and predicted potential transcription factors for* Olfr2* using the PROMO and GeneCards databases. Single-cell sequencing results indicated an upregulation of transcription factors under EHS conditions compared to the NC group (Fig. 7A). We further validated the expression of these transcription factors in TIM-4^+^ smRTM under io+hs conditions using qPCR (Fig. S10A). Next, we used siRNA to knock down the expression of transcription factors *Jund*, *Mga*,* Nr4a1*, *Klf6*, *Jun*, *Fosb*, and *Atf3*. The qPCR results showed that knocking down the transcription factor *Jund* had the most significant impact on reducing *Olfr2* expression (Fig. 7B and S10B).

To investigate JunD binding on the *Olfr2* promoter, we predicted sites using Jaspar. We then synthesized truncation plasmids covering each predicted site and mutants with site mutations (Fig. 7C). The results indicated that the JunD transcription factor binds to the predicted binding sites on the* Olfr2* promoter (Fig. 7D, E). ChIP-qPCR experiments further confirmed this interaction, demonstrating Jund's interaction with the predicted region, and the enrichment signal increased after io + hs treatment (Fig. 7F, G). Additionally, genetic deletion or pharmacological inhibition of HMOX1 markedly reduced the expression of the transcription factor JunD, whereas pharmacological activation of HMOX1 with CoPP significantly increased JunD expression. Notably, co-treatment with the ferroptosis inhibitors Fer-1 or Lip-1 effectively reversed the CoPP-induced upregulation of JunD (Fig. 7H, I). Additionally, knocking down* Jund* resulted in reduced NLRP3 activation, leading to decreased cleaved Caspase-1 expression (Fig. 7J), and reduced secretion of IL-1β in the cell supernatant (Fig. 7K). These findings present evidence that the transcription factor JunD plays a pivotal role in regulating *Olfr2* expression in TIM-4^+^ smRTM. Ferroptosis in TIM-4^+^ smRTMs, Chiefly mediated by HMOX1, promotes the upregulation of JunD.

## 4. Discussion

This study identifies ferroptosis in TIM-4⁺ skeletal muscle resident tissue macrophages (smRTMs) as a mechanistic driver of rhabdomyolysis (RM) during exertional heatstroke (EHS). While EHS is known to cause severe muscle damage and systemic inflammation, the cellular mediators responsible for propagating muscle injury have remained incompletely defined 28. EHS-induced RM has recently been linked to lipid peroxidation and ferroptotic pathways, including ACSL4-mediated lipid remodeling, which directly contributes to RM development following EHS in murine models 29.

smRTMs are strategically located at muscle fiber interfaces, where they sense damage-associated molecular patterns and initiate inflammatory responses 6, 30-32. In contrast to their well-established roles in tissue repair and homeostasis, our findings demonstrate that under EHS conditions, TIM-4⁺ smRTMs undergo HMOX1-dependent ferroptosis, thereby amplifying inflammation and muscle injury. This observation is consistent with accumulating evidence that ferroptosis is tightly linked to immune dysregulation, whereby ferroptotic immune cells release lipid peroxidation products and damage-associated signals that shape inflammatory responses 33. Recent studies have further highlighted TIM-4⁺ tissue-resident macrophages as key regulators of apoptotic cell clearance, tissue remodeling, and injury-associated inflammation, and have demonstrated that ferroptosis can occur in resident macrophage populations under oxidative or metabolic stress, thereby modulating their inflammatory potential 34-37. These findings underscore the relevance of investigating ferroptosis in TIM-4⁺ smRTMs during exertional heatstroke.

Although we refer to these cells as resident tissue macrophages based on their tissue localization and phenotypic characteristics, the precise definition of tissue-resident macrophages remains an area of active debate 38. As F4/80 expression alone is insufficient to unequivocally define tissue residency, and embryonically derived macrophages can be progressively replaced by myeloid-derived CD11b⁺ cells during aging 39-42. In this study, our definition of tissue-resident/mature macrophages was based on a combination of markers (CD45⁺CD11b⁺F4/80⁺CD64⁺), tissue localization, and transcriptional profiling, rather than reliance on a single surface marker 42. Although macrophage plasticity blurs the boundary between resident and infiltrating populations, our central conclusion-that the TIM-4⁺ macrophage subset exhibits heightened susceptibility to ferroptosis under EHS conditions-remains unaffected by nomenclature.

Ferroptosis is driven by iron-dependent lipid peroxidation and is mechanistically distinct from apoptosis, necroptosis, and other regulated cell death modalities 43. Through its dependence on iron and reactive lipid species, ferroptosis can engage in feed-forward interactions with inflammatory cascades, thereby magnifying tissue injury during acute stress 44. This contrasts with apoptotic or necrotic processes, which do not directly involve iron-catalyzed lipid radical formation, underscoring the unique contribution of ferroptosis to inflammation 11. In macrophages, ferroptosis has been implicated in diverse pathological contexts, including atherosclerosis and autoimmune diseases, where it disrupts immune homeostasis and promotes pro-inflammatory signaling 45, 46.

Heme oxygenase-1 (HMOX1) functions as a stress-inducible heme-degrading enzyme, yielding biliverdin, carbon monoxide, and labile iron 47. Although HMOX1 often exerts cytoprotective effects under physiological conditions, excessive or dysregulated HMOX1 expression under stress can elevate intracellular iron levels, thereby promoting lipid peroxidation and ferroptosis 48. Prior studies in macrophages, including alveolar macrophages, have shown that altered HMOX1 expression reshapes iron homeostasis and shifts macrophage function from tissue repair toward pro-inflammatory responses 10, 49. Our findings extend this paradigm to skeletal muscle-resident macrophages, demonstrating that HMOX1-driven ferroptosis in TIM-4⁺ smRTMs serves as a context-dependent amplifier of inflammatory muscle injury during EHS.

A key mechanistic insight of this study is the link between ferroptosis and inflammasome activation mediated by olfactory receptor 2 (Olfr2) 22. Olfr2 was initially characterized in vascular macrophages as a sensor of lipid aldehydes such as octanal that activates NLRP3 inflammasomes and drives IL-1β production 21, but its role in skeletal muscle-resident macrophages had not been explored. Here, we show that lipid peroxidation products generated during ferroptosis upregulate *Olfr2* expression in TIM-4⁺ smRTMs, and that JunD-dependent transcriptional regulation contributes to this process. Activation of the Olfr2-NLRP3 axis promotes IL-1β release, thereby exacerbating RM. This mechanism illustrates how metabolic by-products of ferroptosis can be directly coupled to innate immune signaling.

Although 4-hydroxynonenal (4-HNE) has been reported to inhibit NLRP3 inflammasome activation under specific conditions, its effects are highly context dependent 50, 51. In our EHS model, circulating 4-HNE concentrations (0.4-0.5 μM) were substantially lower than levels previously reported to modulate NLRP3 activity, and exogenous 4-HNE did not measurably alter inflammasome activation in TIM-4⁺ smRTMs under io + hs conditions. In contrast, octanal—but not 4-HNE or MDA—emerged as the dominant lipid aldehyde enhancing NLRP3 activation in this subset, supporting the concept of aldehyde specificity rather than a uniform effect of lipid peroxidation products.

Clinically, RM is a major determinant of poor outcomes in EHS patients. While current management emphasizes rapid cooling and supportive care, inflammatory processes often persist after resolution of hyperthermia. Our findings suggest that targeting ferroptosis or the Olfr2-NLRP3 axis may help interrupt this secondary inflammatory amplification. Although ferroptosis inhibitors such as ferrostatin-1 have shown protective effects in preclinical models, future studies will be required to assess how long-term modulation of ferroptosis influences muscle regeneration and systemic immune balance. Additional investigation of other macrophage subsets and recruited monocyte-derived populations may further refine our understanding of immune regulation in EHS.

## Conclusions

**Figure 8:**
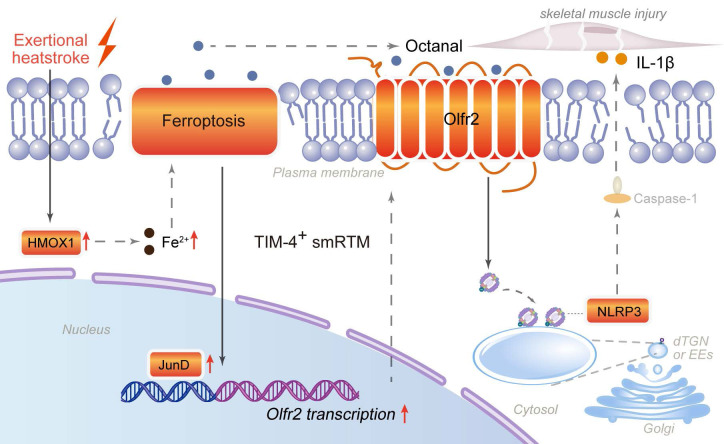
Mechanism diagram of TIM-4^+^ skeletal muscle resident macrophages (smRTMs) ferroptosis-mediated rhabdomyolysis in exertional heatstroke.

Under exertional heatstroke conditions, HMOX1-driven ferroptosis in T cell membrane protein 4-positive skeletal muscle resident tissue macrophages promotes octanal production and induces JunD-dependent transcription of olfactory receptor 2. Enhanced Olfr2 signaling activates the NLRP3 inflammasome and promotes IL-1β release, thereby accelerating skeletal muscle injury and rhabdomyolysis. These findings expand the molecular framework of EHS-associated RM and highlight ferroptosis-inflammasome crosstalk in resident macrophages as a potential target for precision diagnosis and therapy (Fig. 8).

## Supplementary Material

Supplementary figures.

## Figures and Tables

**Table 1:** Quantitative real-time PCR primers.

Gene	Forward	Reverse
*Hmox1*	5′-AAGCCGAGAATGCTGAGTTCA-3′	5′-GCCGTGTAGATATGGTACAAGGA-3′
*Gapdh*	5′- AGGTCGGTGTGAACGGATTTG-3′	5′- TGTAGACCATGTAGTTGAGGTCA-3′
*Ptgs2*	5′-TTCAACACACTCTATCACTGGC-3′	5′-AGAAGCGTTTGCGGTACTCAT-3′
*Cebpb*	5′-GGTTTCGGGACTTGATGCAAT-3′	5′-CGCAGGAACATCTTTAAGTGATTAC-3′
*Olfr2*	5′-ATGGAGCGAAGGAACCACAC-3′	5′-CCAACACGTAGGCCAACAGA-3′
*Mga*	5′-GCTCCGATCAAGAAGGGAATAG-3′	5′-AACTGAGGAACTTCATGTGTCG-3′
*Cebpg*	5′-AGCGGCTTACAGCAGGTTC-3′	5′-GGCGGTATTCGTCACTATTCC-3′
*Nr4a2*	5′-GTGTTCAGGCGCAGTATGG-3′	5′-TGGCAGTAATTTCAGTGTTGGT-3′
*Junb*	5′-TCACGACGACTCTTACGCAG-3′	5′-CCTTGAGACCCCGATAGGGA-3′
*Jun*	5′-CCTTCTACGACGATGCCCTC-3′	5′-GGTTCAAGGTCATGCTCTGTTT-3′
*Irf2*	5′-AATTCCAATACGATACCAGGGCT-3′	5′-GAGCGGAGCATCCTTTTCCA-3′
*Fosb*	5′-CCTCCGCCGAGTCTCAGTA-3′	5′-CCTGGCATGTCATAAGGGTCA-3′
*Crem*	5′-ATGTCTTGAAAATCGTGTGGCT-3′	5′-TGGCAATAAAGGTCTTTGAGGG-3′
*Klf6*	5′-GTTTCTGCTCGGACTCCTGAT-3′	5′-TTCCTGGAAGATGCTACACATTG-3′
*Spi1*	5′-ATGTTACAGGCGTGCAAAATGG-3′	5′-TGATCGCTATGGCTTTCTCCA-3′
*Atf3*	5′-GAGGATTTTGCTAACCTGACACC-3′	5′-TTGACGGTAACTGACTCCAGC-3′
*Nr4a1*	5′-GAGTTCGGCAAGCCTACCAT-3′	5′-GTGTACCCGTCCATGAAGGTG-3′
*Jund*	5′-GAAACGCCCTTCTATGGCGA-3′	5′-CAGCGCGTCTTTCTTCAGC-3′
*Gpx4*	5′-GATGGAGCCCATTCCTGAACC-3′	5′-CCCTGTACTTATCCAGGCAGA-3′
*Sat1*	5′- GAGAACACCCCTTCTACCACT-3′	5′- GCCTCTGTAATCACTCATCACGA-3′
*Ncoa4*	5′- GAAAAGAGGCTATATCCAGGTGC-3′	5′- AAGAAGCCACTCACTCAGAGA-3′
*Acsl4*	5′- CCTGAGGGGCTTGAAATTCAC-3′	5′- GTTGGTCTACTTGGAGGAACG-3′
*Tp53*	5′- CTCTCCCCCGCAAAAGAAAAA-3′	5′- CGGAACATCTCGAAGCGTTTA-3′
*Tfrc*	5′- GTTTCTGCCAGCCCCTTATTAT-3′	5′- GCAAGGAAAGGATATGCAGCA-3′
*Ctsb*	5′- CAGGCTGGACGCAACTTCTAC-3′	5′- TCACCGAACGCAACCCTTC-3′
*Vdac2*	5′- CTCCACCCTATGCTGACCTC-3′	5′- CCCGCTAACTTTACCAGTGTCT-3′
*Acsl1*	5′- TGCCAGAGCTGATTGACATTC-3′	5′- GGCATACCAGAAGGTGGTGAG-3′
*Slc7a11*	5′- GGCACCGTCATCGGATCAG-3′	5′- CTCCACAGGCAGACCAGAAAA-3′
*Prdx6*	5′- CGCCAGAGTTTGCCAAGAG-3′	5′- TCCGTGGGTGTTTCACCATTG-3′
*Acsl3*	5′- AACCACGTATCTTCAACACCATC-3′	5′- AGTCCGGTTTGGAACTGACAG-3′
*Slc40a1*	5′-ACCAAGGCAAGAGATCAAACC-3′	5′-AGACACTGCAAAGTGCCACAT-3′
*Fth1*	5′-CAAGTGCGCCAGAACTACCA-3′	5′-GCCACATCATCTCGGTCAAAA-3′

## References

[1] Laitano O, Oki K, Leon LR (2021). The Role of Skeletal Muscles in Exertional Heat Stroke Pathophysiology. Int J Sports Med.

[2] Warren JD, Blumbergs PC, Thompson PD (2002). Rhabdomyolysis: a review. Muscle Nerve.

[3] O'Connor FG, Grunberg NE, Harp JB, Duster PA (2020). Exertion-Related Illness: The Critical Roles of Leadership and Followership. Curr Sports Med Rep.

[4] Douma MJ, Aves T, Allan KS, Bendall JC, Berry DC, Chang WT (2020). First aid cooling techniques for heat stroke and exertional hyperthermia: A systematic review and meta-analysis. Resuscitation.

[5] Lim CL (2018). Heat Sepsis Precedes Heat Toxicity in the Pathophysiology of Heat Stroke-A New Paradigm on an Ancient Disease. Antioxidants (Basel).

[6] Uderhardt S, Martins AJ, Tsang JS, Lammermann T, Germain RN (2019). Resident Macrophages Cloak Tissue Microlesions to Prevent Neutrophil-Driven Inflammatory Damage. Cell.

[7] Davies LC, Jenkins SJ, Allen JE, Taylor PR (2013). Tissue-resident macrophages. Nat Immunol.

[8] Arango Duque G, Descoteaux A (2014). Macrophage cytokines: involvement in immunity and infectious diseases. Front Immunol.

[9] Goldsmith CA, Frevert C, Imrich A, Sioutas C, Kobzik L (1997). Alveolar macrophage interaction with air pollution particulates. Environ Health Perspect.

[10] Li Y, Yang Y, Guo T, Weng C, Yang Y, Wang Z (2023). Heme oxygenase-1 determines the cell fate of ferroptotic death of alveolar macrophages in COPD. Front Immunol.

[11] Dixon SJ, Lemberg KM, Lamprecht MR, Skouta R, Zaitsev EM, Gleason CE (2012). Ferroptosis: an iron-dependent form of nonapoptotic cell death. Cell.

[12] Kapralov AA, Yang Q, Dar HH, Tyurina YY, Anthonymuthu TS, Kim R (2020). Redox lipid reprogramming commands susceptibility of macrophages and microglia to ferroptotic death. Nat Chem Biol.

[13] Bleriot C, Chakarov S, Ginhoux F (2020). Determinants of Resident Tissue Macrophage Identity and Function. Immunity.

[14] Shi H, Wang X, Sloas C, Gerlach B, Yurdagul A Jr, Moore MP (2025). Impaired TIM4-mediated efferocytosis by liver macrophages contributes to fibrosis in metabolic dysfunction-associated steatohepatitis. Sci Transl Med.

[15] Ni J, Zhang R, Pu Y, He Y, Hu W, Su L (2026). Targeting Myeloid FoxO1 Ameliorates Sepsis-induced Intestinal Injury by Modulating Tim4(+) Macrophage Glycolysis. Int J Biol Sci.

[16] Hirao H, Kageyama S, Nakamura K, Kadono K, Kojima H, Siyuan Y (2023). Recipient TIM4 signaling regulates ischemia reperfusion-induced ER stress and metabolic responses in liver transplantation: from mouse-to-human. Front Transplant.

[17] Horn P, Tacke F (2024). Metabolic reprogramming in liver fibrosis. Cell Metab.

[18] Feng Z, Meng F, Huo F, Zhu Y, Qin Y, Gui Y (2024). Inhibition of ferroptosis rescues M2 macrophages and alleviates arthritis by suppressing the HMGB1/TLR4/STAT3 axis in M1 macrophages. Redox Biol.

[19] Chen X, Wang J, Yang P, Liu HY, Zhong S, Lu C (2024). SENP3 sensitizes macrophages to ferroptosis via de-SUMOylation of FSP1. Redox Biol.

[20] Rizzo WB (2014). Fatty aldehyde and fatty alcohol metabolism: review and importance for epidermal structure and function. Biochim Biophys Acta.

[21] Orecchioni M, Kobiyama K, Winkels H, Ghosheh Y, McArdle S, Mikulski Z (2022). Olfactory receptor 2 in vascular macrophages drives atherosclerosis by NLRP3-dependent IL-1 production. Science.

[22] Wang C, Andreasson KI (2022). Odorant receptors in macrophages: potential targets for atherosclerosis. Trends Immunol.

[23] Aktories P, Petry P, Glatz P, Andrieux G, Oschwald A, Botterer H (2022). An improved organotypic cell culture system to study tissue-resident macrophages *ex vivo*. Cell Rep Methods.

[24] Villalta SA, Nguyen HX, Deng B, Gotoh T, Tidball JG (2009). Shifts in macrophage phenotypes and macrophage competition for arginine metabolism affect the severity of muscle pathology in muscular dystrophy. Hum Mol Genet.

[25] Sun Y, Chen P, Zhai B, Zhang M, Xiang Y, Fang J (2020). The emerging role of ferroptosis in inflammation. Biomed Pharmacother.

[26] Remmerie A, Martens L, Thone T, Castoldi A, Seurinck R, Pavie B (2020). Osteopontin Expression Identifies a Subset of Recruited Macrophages Distinct from Kupffer Cells in the Fatty Liver. Immunity.

[27] Swanson KV, Deng M, Ting JP (2019). The NLRP3 inflammasome: molecular activation and regulation to therapeutics. Nat Rev Immunol.

[28] Bouchama A, Abuyassin B, Lehe C, Laitano O, Jay O, O'Connor FG (2022). Classic and exertional heatstroke. Nat Rev Dis Primers.

[29] He S, Li R, Peng Y, Wang Z, Huang J, Meng H (2022). ACSL4 contributes to ferroptosis-mediated rhabdomyolysis in exertional heat stroke. J Cachexia Sarcopenia Muscle.

[30] Wang X, Zhao W, Ransohoff RM, Zhou L (2018). Infiltrating macrophages are broadly activated at the early stage to support acute skeletal muscle injury repair. J Neuroimmunol.

[31] Babaeijandaghi F, Cheng R, Kajabadi N, Soliman H, Chang CK, Smandych J (2022). Metabolic reprogramming of skeletal muscle by resident macrophages points to CSF1R inhibitors as muscular dystrophy therapeutics. Sci Transl Med.

[32] Dick SA, Wong A, Hamidzada H, Nejat S, Nechanitzky R, Vohra S (2022). Three tissue resident macrophage subsets coexist across organs with conserved origins and life cycles. Sci Immunol.

[33] Chen X, Kang R, Kroemer G, Tang D (2021). Ferroptosis in infection, inflammation, and immunity. J Exp Med.

[34] Chen Y, Fang ZM, Yi X, Wei X, Jiang DS (2023). The interaction between ferroptosis and inflammatory signaling pathways. Cell Death Dis.

[35] Liu T, Huang Y, Wang Y, Shen H (2025). Disrupting the immune homeostasis: the emerging role of macrophage ferroptosis in autoimmune diseases. Int Immunopharmacol.

[36] Ismahil MA, Zhou G, Rajasekar S, Gao M, Bansal SS, Patel B (2025). Splenic CD169(+)Tim4(+) Marginal Metallophilic Macrophages Are Essential for Wound Healing After Myocardial Infarction. Circulation.

[37] Huh JY, Kim JB (2021). TIM4(+) adipose tissue-resident macrophages: new modulators of adiposity. Nat Rev Endocrinol.

[38] Li J, Xiao C, Li C, He J (2025). Tissue-resident immune cells: from defining characteristics to roles in diseases. Signal Transduct Target Ther.

[39] Arnold L, Henry A, Poron F, Baba-Amer Y, van Rooijen N, Plonquet A (2007). Inflammatory monocytes recruited after skeletal muscle injury switch into antiinflammatory macrophages to support myogenesis. J Exp Med.

[40] Lazarov T, Juarez-Carreno S, Cox N, Geissmann F (2023). Physiology and diseases of tissue-resident macrophages. Nature.

[41] Ginhoux F, Guilliams M (2016). Tissue-Resident Macrophage Ontogeny and Homeostasis. Immunity.

[42] Wang X, Sathe AA, Smith GR, Ruf-Zamojski F, Nair V, Lavine KJ (2020). Heterogeneous origins and functions of mouse skeletal muscle-resident macrophages. Proc Natl Acad Sci U S A.

[43] Ru Q, Li Y, Chen L, Wu Y, Min J, Wang F (2024). Iron homeostasis and ferroptosis in human diseases: mechanisms and therapeutic prospects. Signal Transduct Target Ther.

[44] Yu Y, Yan Y, Niu F, Wang Y, Chen X, Su G (2021). Ferroptosis: a cell death connecting oxidative stress, inflammation and cardiovascular diseases. Cell Death Discov.

[45] Jinson S, Zhang Z, Lancaster GI, Murphy AJ, Morgan PK (2025). Iron, lipid peroxidation, and ferroptosis play pathogenic roles in atherosclerosis. Cardiovasc Res.

[46] Jiang Q, Wan R, Jiang J, Li T, Li Y, Yu S (2025). Interaction between macrophages and ferroptosis: Metabolism, function, and diseases. Chin Med J (Engl).

[47] Vitek L, Hinds TD Jr, Stec DE, Tiribelli C (2023). The physiology of bilirubin: health and disease equilibrium. Trends Mol Med.

[48] Menon AV, Liu J, Tsai HP, Zeng L, Yang S, Asnani A (2022). Excess heme upregulates heme oxygenase 1 and promotes cardiac ferroptosis in mice with sickle cell disease. Blood.

[49] Vijayan V, Wagener F, Immenschuh S (2018). The macrophage heme-heme oxygenase-1 system and its role in inflammation. Biochem Pharmacol.

[50] Hsu CG, Chavez CL, Zhang C, Sowden M, Yan C, Berk BC (2022). The lipid peroxidation product 4-hydroxynonenal inhibits NLRP3 inflammasome activation and macrophage pyroptosis. Cell Death Differ.

[51] Kauppinen A, Niskanen H, Suuronen T, Kinnunen K, Salminen A, Kaarniranta K (2012). Oxidative stress activates NLRP3 inflammasomes in ARPE-19 cells-implications for age-related macular degeneration (AMD). Immunol Lett.

