# Supplementary material for: TIM-4+ skeletal muscle Resident Tissue Macrophages Ferroptosis mediated Rhabdomyolysis in Exertional Heatstroke
Source: Int J Biol Sci. 2026 Mar 17;22(7):3432–50. doi: 10.7150/ijbs.114815 (PMC13085888; doi:10.7150/ijbs.114815)
Supplement: Supplementary file 1 — Supplementary figures. [file ijbsv22p3432s1.pdf]

Supplementary figures

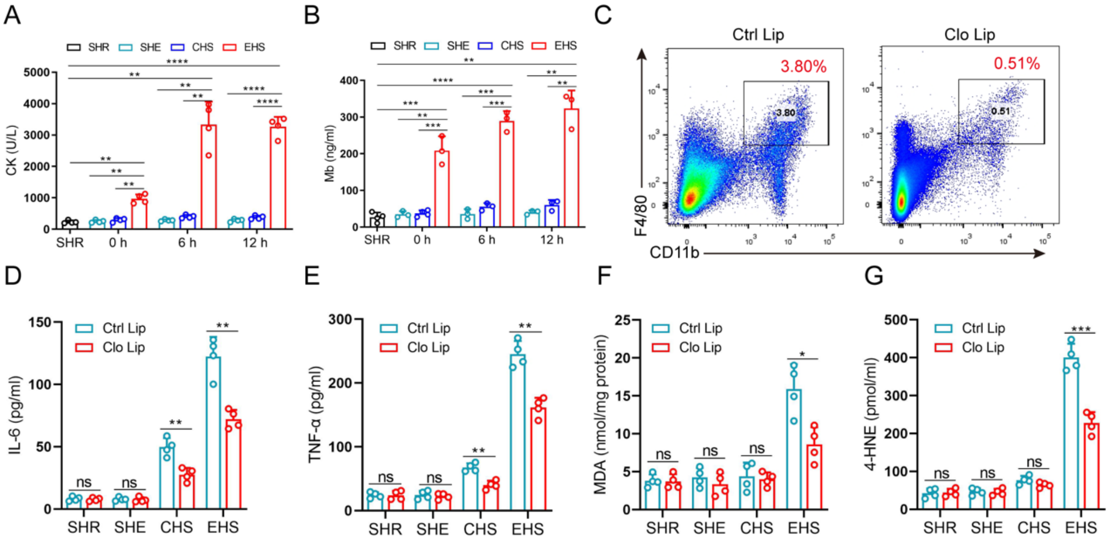

Fig. S1 The role of smRTM ferroptosis in promoting skeletal muscle injury in EHS mice (A-B) Plasma CK and Mb levels were measured (n=3-5 mice/group). **(C)** Flow cytometry was used to analyze the change in the number of smRTM after Clo Lip treatment. **(D-E)** The level of IL-6 and TNF-α. **(F-G)** The levels of MDA and 4-HNE at 6 h were assayed (n = 3-5 mice/group). Data are mean ± SEM. **P<0.05*, ***P<0.01*, ****P<0.001*, *****P<0.0001* by Student's t-test.

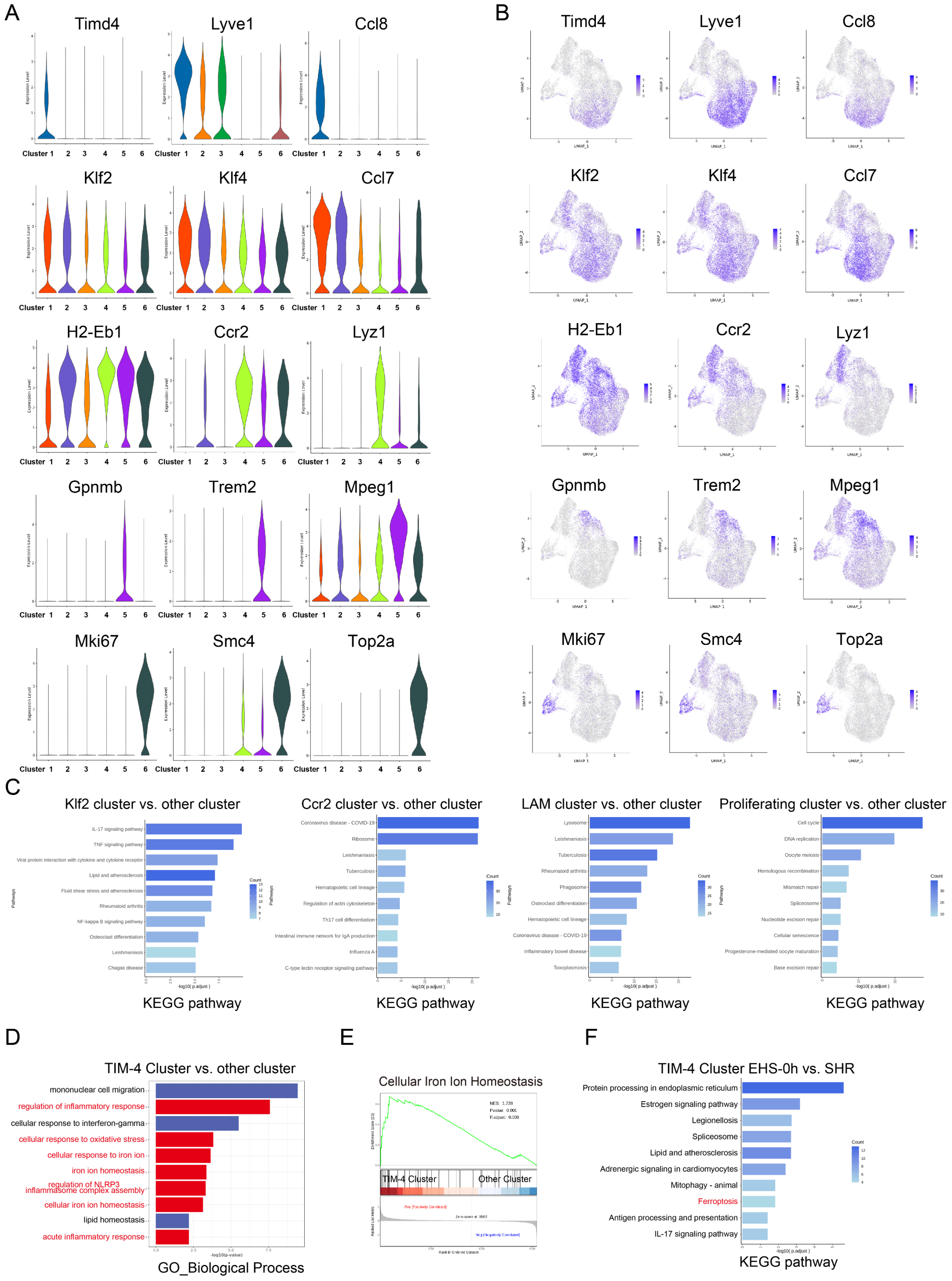

Fig. S2. Single-cell RNA-seq reveals macrophage heterogeneity and dynamic changes after EHS. (A–B) Violin plots **(A)** and feature plots **(B)** show marker gene expression across clusters: *Timd4*, *Lyve1*, *Ccl8* (TIM-4$^+$ cluster); *Klf2*, *Klf4*, *Ccl7* (Klf2$^+$ cluster); *H2-Eb1*, *Ccr2*, *Lyz1* (Ccr2$^+$ cluster); *Gpnmb*, *Trem2*, *Mpeg1* (LAM

cluster); and *Mki67*, *Smc4*, *Top2a* (proliferating cluster). **(C)** KEGG enrichment of upregulated genes in Klf2, Ccr2, LAM, and proliferating clusters. **(D)** GO analysis of DEGs in the TIM-4$^+$ cluster highlights roles in inflammation and iron homeostasis. **(E)** GSEA confirms enrichment of iron ion homeostasis pathways in TIM-4$^+$ macrophages. **(F)** KEGG pathway analysis comparing TIM-4$^+$ cells from EHS-0 h and SHR reveals enrichment in ferroptosis-related pathways.

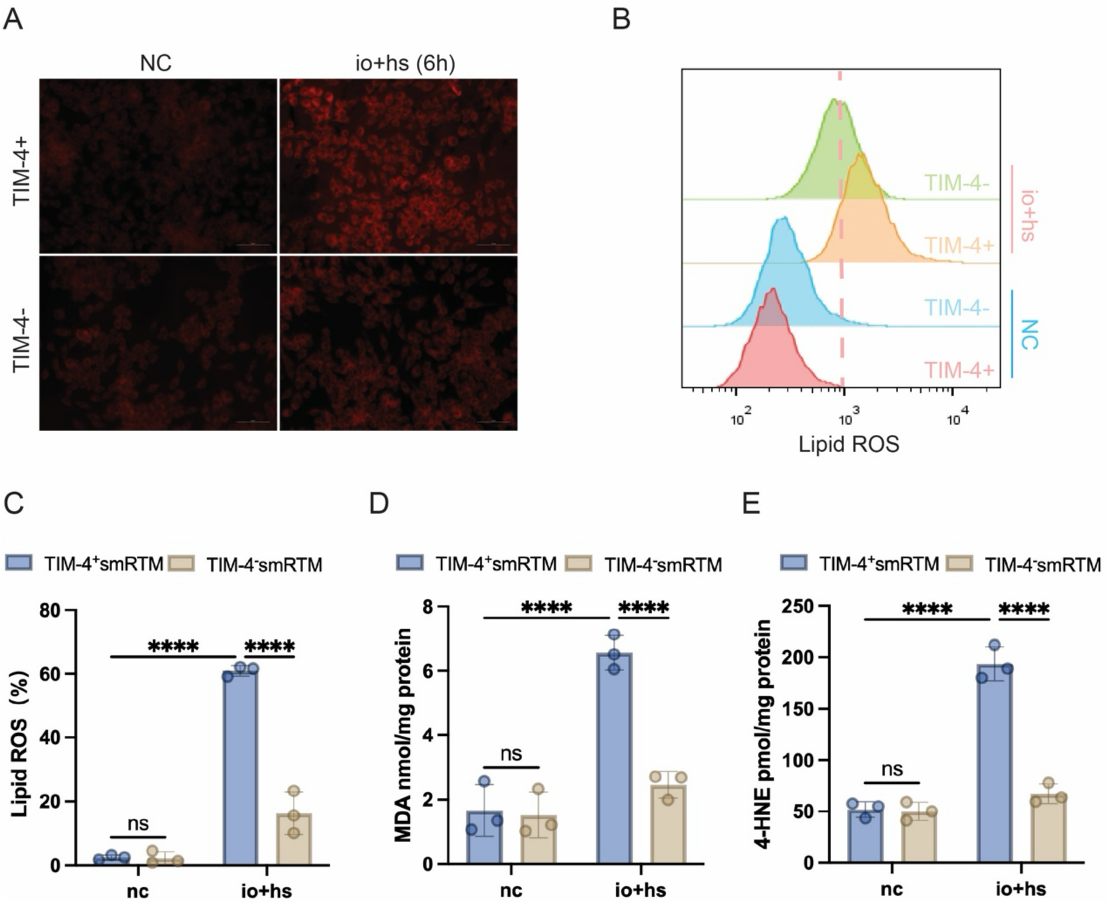

Fig. S3. TIM-4$^+$ smRTMs exhibit elevated lipid peroxidation under EHS conditions. (A–B) Representative images **(A)** and flow cytometry **(B)** showing increased lipid ROS in TIM-4$^+$ smRTMs compared to TIM-4$^-$ cells after io+hs. **(C–E)** Quantification of lipid ROS **(C),** MDA **(D)**, and 4-HNE **(E)** levels confirms enhanced

lipid peroxidation in TIM-4+ smRTMs under EHS. Data are mean ± SEM. **P<0.05*, ***P<0.01*, ****P<0.001*, *****P<0.0001* by Student's t-test.

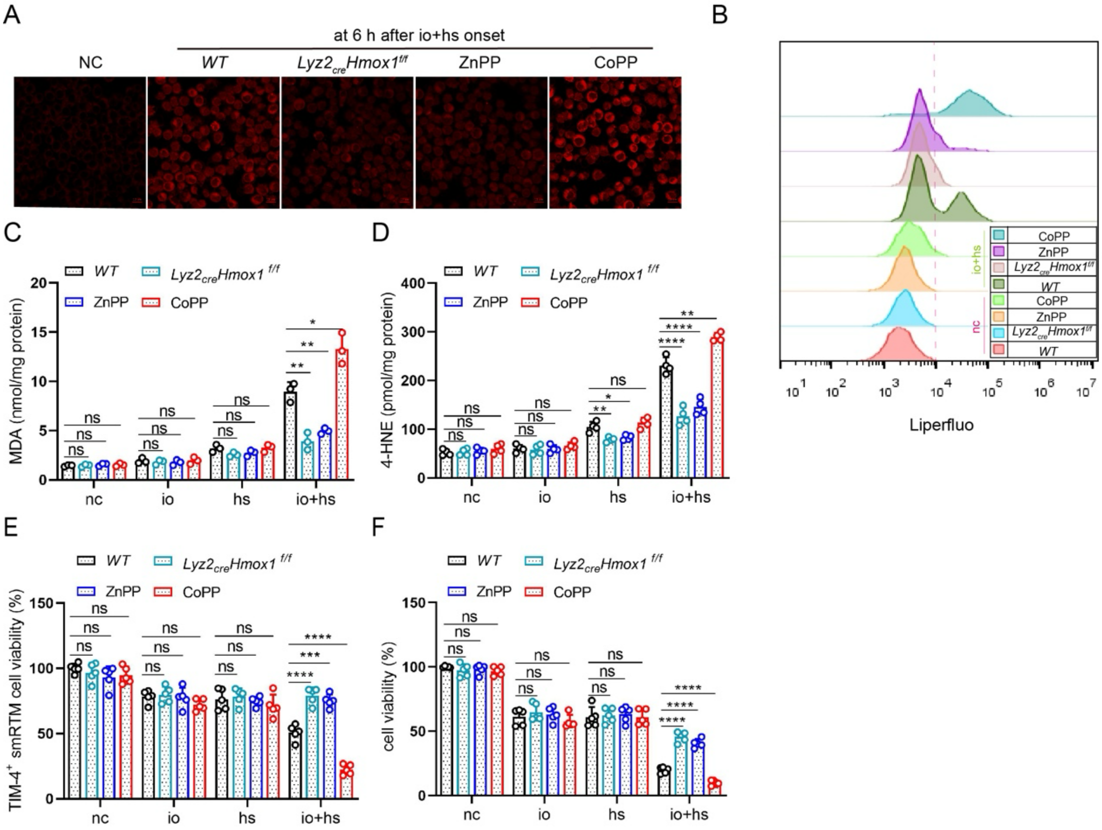

Fig. S4 HMOX1 induced TIM-4$^+$ smRTM ferroptosis *in vitro* **(A)** Representative images of FerroOrange staining in TIM-4$^+$ smRTM following pretreatment with ZnPP, CoPP, or *Hmox1* knockout at 6 h post io+hs exposure. **(B)** Flow cytometry was employed to analyze lipid peroxidation (n = 3). **(C-D)** The levels of MDA and 4-HNE were assayed (n = 3). **(E)** Cell viability was assessed **(F)** Cell viability was assessed in C2C12 cell co-cultured with pretreated TIM-4$^+$ smRTM. Data are mean ± SEM. **P<0.05*, ***P<0.01*, ****P<0.001*, *****P<0.0001* by Student's t-test.

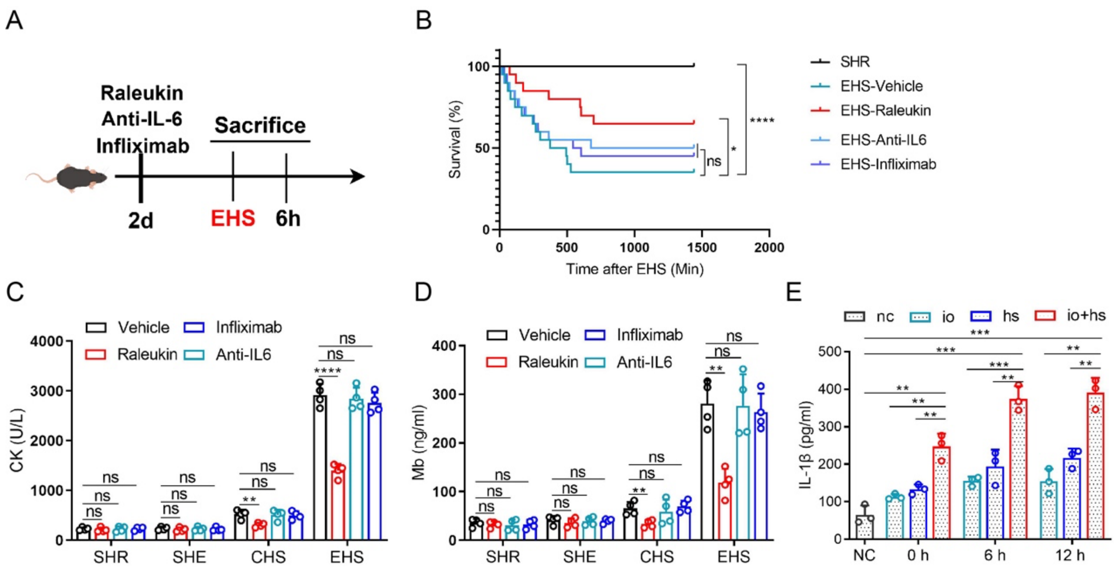

Fig. S5 Inhibition of IL-1β secretion could reduce skeletal muscle injury (A) Schematic illustration of Raleukin, Anti-IL-6, and Infliximab in modulating inflammatory cytokine activity. **(B)** Survival curves are shown for mice pretreated with Raleukin, Anti-IL-6, and Infliximab, followed by EHS exposure (n =20 mice/group). **(C-D)** Detection of CK and Mb in plasma of pretreated mice (n=3-5 mice/group). **(E)** Detection of IL-1β content in cell supernatant. Data are mean ± SEM. **P<0.05*, ***P<0.01*, ****P<0.001*, *****P<0.0001* by Student's t-test.

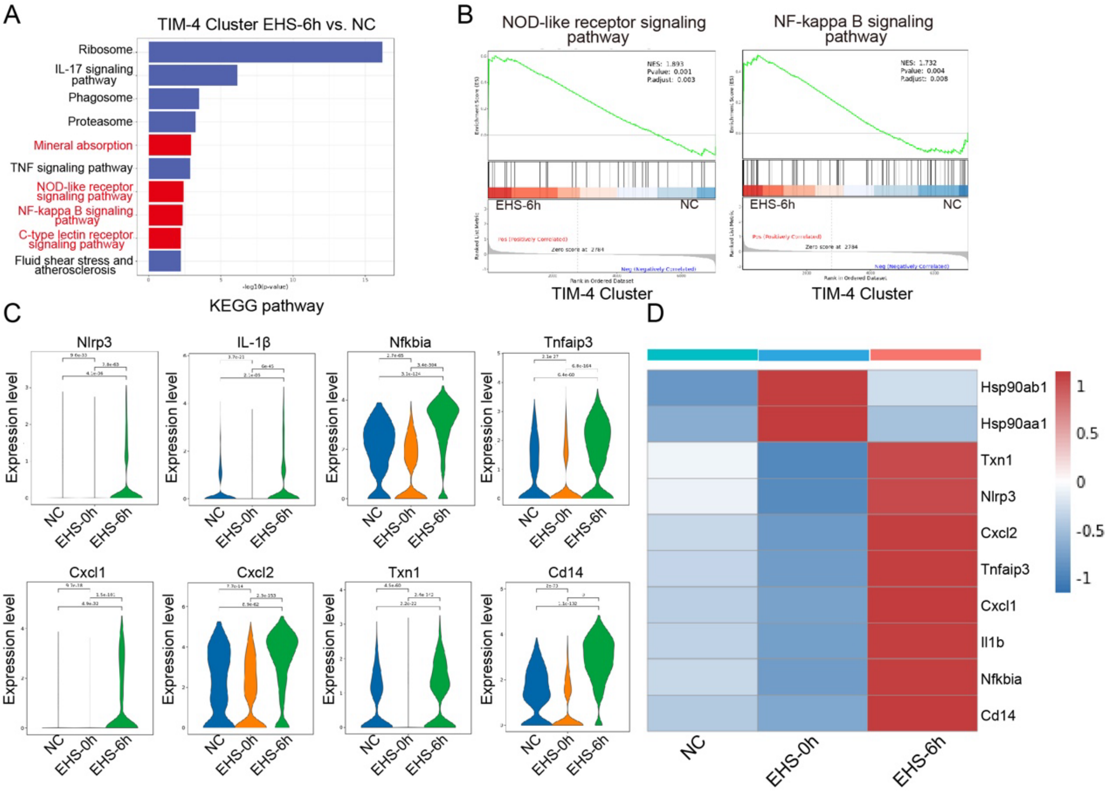

Fig. S6 Single-cell RNA sequencing reveals NLRP3 inflammasome activation under EHS conditions (A) KEGG enrichment analysis was performed to display the top 10 enriched pathways in EHS-recovered mice at 6 h compared to SHR mice. **(B)** Gene set enrichment analysis (GSEA) was conducted to analyze EHS-recovered mice at 6 h and SHR mice. **(C-D)** Violin plots **(C)** and Heat maps **(D)** were used to visualize the expression of genes associated with the NOD-receptor signaling pathway under EHS conditions.

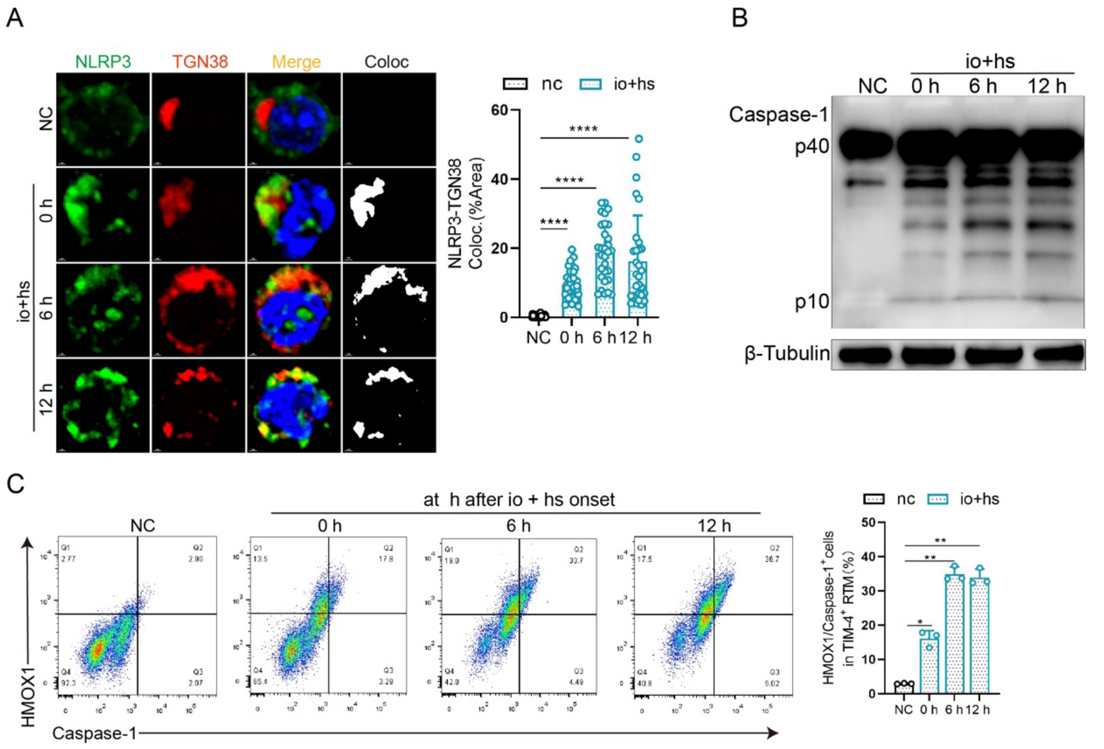

Fig. S7 The relationship between EHS and NLRP3 activation (A) Representative images of immunofluorescence staining in TIM-4$^+$ smRTM and statistical analysis of co-localization of NLRP3 and TGN38. **(B)** Western Blotting was employed to analyze Caspase-1 expression in TIM-4$^+$ smRTM. **(C)** Flow cytometry was used to analyze the proportion of Hmox1$^+$/Caspase-1$^+$ cells in TIM-4$^+$ smRTM (n = 3). Data are mean ± SEM. **P<0.05*, ***P<0.01*, ****P<0.001*, *****P<0.0001* by Student's t-test.

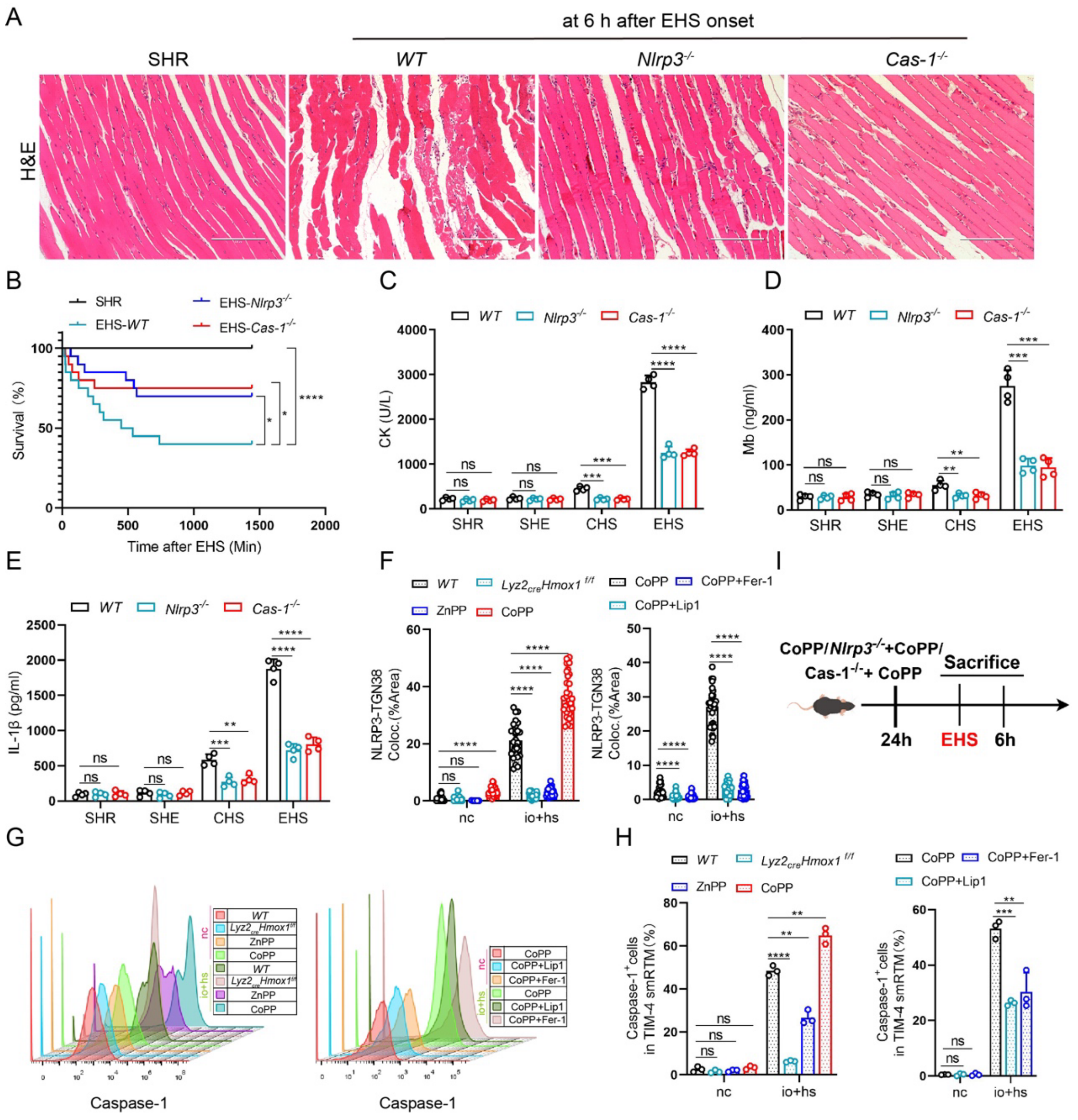

Fig. S8 NLRP3 inflammasome activation promotes skeletal muscle injury induced by EHS (A) Representative HE-staining shows skeletal muscle sections from *Nlrp3*$^{-/-}$ mice or *Cas-1*$^{-/-}$ mice, followed by EHS. **(B) S**urvival curves of *Nlrp3*$^{-/-}$ mice or *Cas-1*$^{-/-}$ mice (n = 20 mice/group). **(C-D)** Measurement of CK and Mb levels (n =3-5 mice/group). Samples were collected at 6 h post EHS onset. **(E)** Plasma IL-1β content were detected (n = 3-5 mice/group). **(F)** Statistical analysis of NLRP3 and TGN38 co-localization in Fig. 5E is presented. **(G-H)** Analysis of Caspase-1 expression in pretreated TIM-4^{+}smRTM and statistical analysis (n = 3). **(I)** Schematic

diagram illustrating NLRP3 inflammasome regulation through *Nlrp3* or *Cas-1* knockout and CoPP. Data are mean ± SEM. *$P<0.05$, **$P<0.01$, ***$P<0.001$, ****$P<0.0001$ by Student's t-test.

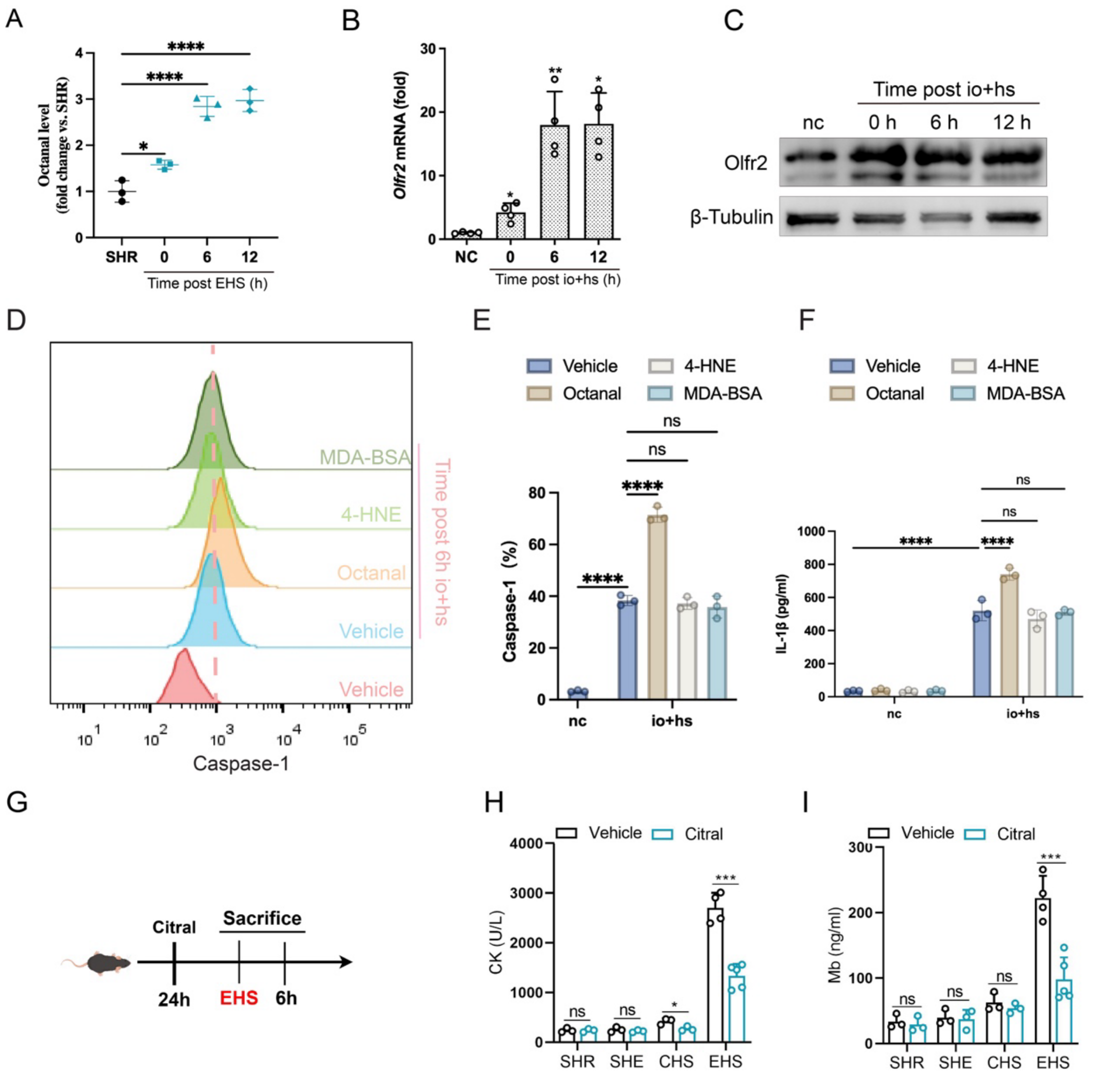

Fig. S9 Octanal promotes NLRP3 inflammasome activation and exacerbates muscle injury under EHS conditions. (A) Octanal levels in plasma were quantified by LC-MS at indicated time points post-EHS. **(B, C)** qPCR **(B)** and Western blot **(C)** showing time-dependent upregulation of the octanal receptor Olfr2 in TIM-4^{+} smRTMs after io+hs stimulation. **(D)** Flow cytometry plots of Caspase-1 activation in

macrophages treated with Octanal, MDA-BSA, or 4-HNE. **(E, F)** Quantification of Caspase-1^{+} cells **(E)** and IL-1β secretion **(F)** following lipid aldehyde stimulation under io+hs. **(G)** Experimental scheme for Citral (an Olfr2 antagonist) administration and EHS induction. **(H, I)** Plasma CK **(H)** and Mb **(I)** levels showing that Citral administration attenuated muscle damage during EHS. Data are presented as mean ± SEM; **P<0.05, **P<0.01, ***P<0.001, ****P<0.0001* by one-way ANOVA or Student's t-test, as appropriate.

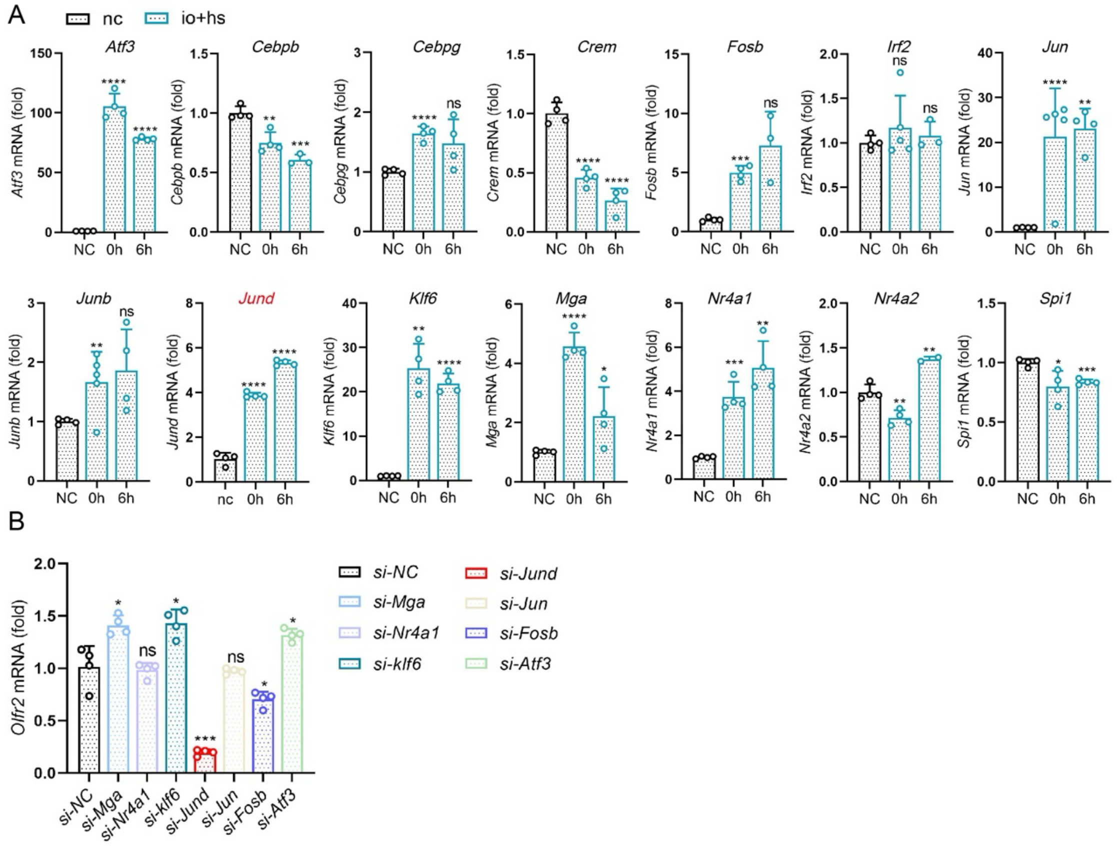

Fig. S10 JunD regulates the transcription of *Olfr2*. (A) qPCR showing the expression of *Atf3*, *Cebpb*, *Cebpg*, *Crem*, *Fosb*, *Irf2*, *Jun*, *Junb*, *Jund*, *Klf6*, *Mga*, *Nr4a1*, *Nr4a2*, and *Spi1* in TIM4^{+} smRTM under io+hs conditions compared to the NC group. **(B)** qPCR analysis of *Olfr2* expression after knocking down *Mga*, *Nr4a1*,

Klf6, *Jund*, *Jun*, *Fosb*, and *Atf3* in TIM-4$^+$ smRTM. Summary data are presented as the mean ± SEM. Significance was calculated using the Student's t-test. ($*P<0.05$, $**P<0.01$, $***P<0.001$, $****P<0.0001$).
